# Test-Retest Reliability of Neural Correlates of Response Inhibition and Error Monitoring: An fMRI Study of a Stop-Signal Task

**DOI:** 10.3389/fnins.2021.624911

**Published:** 2021-01-28

**Authors:** Ozlem Korucuoglu, Michael P. Harms, Serguei V. Astafiev, Semyon Golosheykin, James T. Kennedy, Deanna M. Barch, Andrey P. Anokhin

**Affiliations:** ^1^Department of Psychiatry, Washington University School of Medicine, St. Louis, MO, United States; ^2^Department of Psychological and Brain Sciences, Washington University, St. Louis, MO, United States

**Keywords:** response inhibition, error monitoring, stop-signal task, fMRI, test-retest reliability, familial influences

## Abstract

Response inhibition (RI) and error monitoring (EM) are important processes of adaptive goal-directed behavior, and neural correlates of these processes are being increasingly used as transdiagnostic biomarkers of risk for a range of neuropsychiatric disorders. Potential utility of these purported biomarkers relies on the assumption that individual differences in brain activation are reproducible over time; however, available data on test-retest reliability (TRR) of task-fMRI are very mixed. This study examined TRR of RI and EM-related activations using a stop signal task in young adults (*n* = 56, including 27 pairs of monozygotic (MZ) twins) in order to identify brain regions with high TRR and familial influences (as indicated by MZ twin correlations) and to examine factors potentially affecting reliability. We identified brain regions with good TRR of activations related to RI (inferior/middle frontal, superior parietal, and precentral gyri) and EM (insula, medial superior frontal and dorsolateral prefrontal cortex). No subcortical regions showed significant TRR. Regions with higher group-level activation showed higher TRR; increasing task duration improved TRR; within-session reliability was weakly related to the long-term TRR; motion negatively affected TRR, but this effect was abolished after the application of ICA-FIX, a data-driven noise removal method.

## Introduction

Response inhibition (RI) and error monitoring (EM) are key component processes of adaptive self-regulation of behavior. RI allows individuals to suppress prepotent or ongoing actions that are no longer goal-appropriate in a changing environment. In everyday life, inhibitory control is crucial for suppressing impulsive reactions that are incompatible with larger or longer-term behavioral goals, including responses triggered by the cues of potential reward such as unhealthy foods, compulsive buying or substance-related cues in addicted individuals. EM involves error detection and processing, which is essential for adaptive adjustments of subsequent behavior.

Deficits in response inhibition and aberrant error processing have been implicated as potential transdiagnostic risk factors in a range of neuropsychiatric disorders and maladaptive behaviors characterized by poor self-control and impulsive responding, including attention deficit hyperactivity disorder (ADHD), substance dependence, and obsessive-compulsive disorder (OCD) (Gorenstein and Newman, 1980; Aron and Poldrack, 2005; Chamberlain and Sahakian, 2007; de Wit et al., 2012; Manoach and Agam, 2013; Anokhin and Golosheykin, 2015). For example, patients with ADHD or OCD show poor response inhibition and altered brain activation compared to healthy controls. While blunted error responses are associated with substance abuse, schizophrenia, Autism Spectrum Disorder, exaggerated error processing is present in anxiety, depression, and OCD (Olvet and Hajcak, 2008; Manoach and Agam, 2013; Anokhin and Golosheykin, 2015).

It has been hypothesized that etiological pathways from genes to “disinhibited” behaviors involve a dysfunction of neural substrates subserving response inhibition, and individual differences in inhibition-related neural activity has been proposed as an intermediate phenotype (endophenotype) mediating genetic influences on inhibitory control (Anokhin et al., 2004). Similarly, neural correlates of error monitoring have been shown to be heritable (Anokhin et al., 2008) and suggested to mediate the pathway between genetic predisposition and disease state, and thus have been recommended as a useful endophenotype for psychiatric disorders (Anokhin et al., 2008; Olvet and Hajcak, 2008).

Identification of neural endophenotypes or biomarkers of RI and EM relies on the assumption that neural correlates of inhibition and error processing are stable traits, i.e., individual differences are reproducible over time. While electrophysiological correlates of RI and EM show good reliability and heritability (Anokhin et al., 2004, 2008, 2017), evidence for reliability of brain activation assessed using task-based functional Magnetic Resonance Imaging (fMRI) has been mixed. Over the past years, there has been increasing concern about the reliability of task-fMRI measures, with initial estimates of test-retest reliability (TRR) of around 0.5 (Bennett and Miller, 2010) giving place to more pessimistic conclusions (Fröhner et al., 2019; Elliott et al., 2020). Most notably, a recent study utilizing two different test-retest samples and a meta-analysis of published data has found that several commonly used fMRI task paradigms fail to produce reliable measures of brain activation (Elliott et al., 2020). Overall, studies suggest that reliability of task-based fMRI measures is highly task- and brain region-specific. Often regions that show the highest reliabilities are not necessarily the regions associated with the cognitive processes targeted by the task (e.g., good reliability of activation in motor regions rather than reward-related brain signal in a reward processing task, Fliessbach et al., 2010). Therefore, TRR has to be established for each individual task and region of interest. Given that fMRI correlates of RI and EM are being increasingly used as putative endophenotypes or biomarkers of risk in studies concerned with individual differences, psychopathology, and genetics, it is essential to establish reliability of these measures to test the implicit assumption that such activations represent stable, trait-like measures.

Reliability of brain functional correlates of RI assessed using fMRI remains unclear, as evidence is mixed and often based on very small samples (Zandbelt et al., 2008; Raemaekers et al., 2012; Buimer et al., 2020). The study of Buimer and colleagues reported lower whole-brain average reliabilities (average reliability = 0.44) of RI than average reliabilities for a set of selected ROIs (average reliability = 0.54), using a Stop Signal Anticipation Task. Raemaekers et al. (2012) assessed inter-session stability of fMRI correlates of RI using a modified version of the Stop Signal Task (SST, a widely used response inhibition task) and found that estimates of the amplitude of the activation were not reliable (no test-retest correlations were reported). Another study by Zandbelt et al. (2008) demonstrated that individual activation levels were highly unstable (no test-retest correlations reported), although group-level spatial activation pattern, and group-wise BOLD signal changes were highly stable. In all three studies, estimates of fMRI reliability were based on small samples (*n* = 17, 21 and 10, respectively) and only estimated short-term TRRs (1 week in all studies). TRR of fMRI correlates of EM is even less clear. We are not aware of published TRR studies, but one study reported high within-session internal consistency reliability (Cronbach alpha > 0.7) in the anterior cingulate cortex (ACC) and medial/middle frontal gyrus for data composed of 6 or more error trials (Steele et al., 2016).

Previous studies of RI-related brain activity using the SST have described a right dominant inhibition network including the inferior frontal gyrus (IFG), middle frontal gyrus (MFG), pre-supplementary motor area (pre-SMA), anterior insula, putamen, dorsolateral prefrontal cortex (DLPFC) and inferior-parietal lobule (Eagle et al., 2008; Swick et al., 2011; Cai et al., 2014; Neta et al., 2015). Erroneous responses (failed inhibition) in the SST were associated with activations in the dorsomedial PFC, dorsal ACC, pre-SMA, left insula, thalamus, and left IFG (de Ruiter et al., 2012; Spunt et al., 2012; Luo et al., 2013; also see Ridderinkhof et al., 2004, for a review; and Neta et al., 2015, for a meta-analysis). However, the extent to which regional brain activations observed in the SST represent stable, reproducible individual differences in brain function is not clear. It is important to establish TRR of activation patterns in response to inhibition and error processing during SST because this task is being increasingly used in fMRI studies concerned with individual differences, psychopathology, genetics, and development, such as the Adolescent Brain Cognitive Development (ABCD) Study, a multi-center long-term study of brain development and child health (Casey et al., 2018).

The purpose of the present study was to estimate the TRR of inhibition and error-related regional brain activation using fMRI data from the SST in a community-based sample composed of young adult monozygotic twins (MZ). Participants performed the task twice with an average interval of 6 months between assessments. The current investigation was part of a broader effort aimed at quantifying fMRI reliabilities across different neurocognitive constructs, identifying good candidate regions (endophenotypes) with both high reliability and familiality for genetic and clinical studies, and factors that affect fMRI reliability (see e.g., our recent report on TRR of neural correlates of risk taking, Korucuoglu et al., 2020).

The inclusion of MZ twins allowed us to estimate familiality (familial transmission) by measuring intrapair twin correlations that arise from both genetic commonality and/or shared environmental influences and can serve as a direct measure of the degree of familial transmission of a trait. Although genetic and shared environmental factors cannot be distinguished using MZ twins alone, MZ correlations serve as an upper limit of heritability and can be used to identify potentially heritable traits. It is important to note that as only stable-trait-like measures can be heritable, TRR can also be regarded as the upper boundary for heritability (McCrae et al., 2011). Therefore, we expected a positive correlation between test-retest and twin correlations.

The second aim of this study was to examine different factors that might affect TRR of task-related regional brain activations. Our previous work (Korucuoglu et al., 2020) as well as other studies (Raemaekers et al., 2007; Caceres et al., 2009) suggests that regions showing high group-level task activation tend to have higher TRRs. Since this relationship may vary as a function of task and specific contrast within the task, we examined the correlation between activation magnitude and its TRR across cortical regions in the SST task. We expected a positive correlation, such that regions showing larger task-related activation will show higher TRR and vice versa. Another factor that could potentially affect TRR is task/scan duration. Resting-state fMRI studies have consistently shown that longer scan duration results in more reliable estimates on functional connectivity (Birn et al., 2013). To examine this possibility in the SST data, we compared TRRs computed using a single 6-min run vs. two consecutive runs. Next, we compared within-session reliability (correlation between the two consecutive runs) and long-term TRR between two scanning sessions separated by 6 months (on average) and tested whether the former correlates with the latter. Motion is a major known problem in fMRI research that can confound (increase or decrease) individual and group differences because motion itself is a stable trait (Engelhardt et al., 2017) and the amount of motion differ across groups (e.g., patients and controls). Therefore, we examined whether the amount of in-scanner motion is associated with within-individual stability of activation magnitude. Finally, we compared reliability estimates before and after the application of a data-driven method for the removal of structured noise, the independent component analysis (ICA) based “FIX” cleanup (Beckmann and Smith, 2005; Griffanti et al., 2014). This method first identifies structured artifacts and non-artifactual components from an fMRI data series at the subject level, and then uses a hierarchical machine learning classifier, which is followed by removal of artifact components (Smith et al., 2013). In the Human Connectome Project dataset, the FIX classifier method has been shown to achieve 99% accuracy.

In summary, the present study addressed the following questions: How reliable are fMRI-measured individual differences in brain activation related to RI and EM? Are these brain activations influenced by familial factors? Do regions with stronger group-level task-related activation show higher TRR and familiality? Does longer scanning duration increase TRR? Does within-session reliability relate to longer-term (between-session) reliability? How does in-scanner motion affect the within-subject stability of estimated activation? Does ICA-FIX artifact removal improve the TRR of brain activations?

## Materials and Methods

### Participants

Fifty-six young adults (32 females, age range: 21–24 years, mean = 23.3, SD = 0.86) participated in the study. Participants were monozygotic (MZ) twins ascertained for the present study through the Missouri Family Registry at the Department of Psychiatry at Washington University School of Medicine (WUSM) as part of a larger longitudinal study - Genetics, Neurocognition, and Adolescent Substance Abuse (GNASA; Anokhin et al., 2017). All 56 participants (including 27 MZ twin pairs) in the present study completed the first MRI scanning session (Time1), and 44 of them (26 females, 19 MZ twin pairs, age range: 21–24 years, mean = 23.3, SD = 0.89 at Time1, age range 22–25 years, mean = 23.96, SD = 0.9 at Time 2) completed a second session approximately 6 months later (Time2, mean (SD) interval 7.9 months (1.70), ranging from 5.7 to 12.0 months). Exclusion criteria included (1) standard MRI contraindications such as non-removable metal in the body, dental braces, excessive weight, claustrophobia, current pregnancy, or difficulty lying supine; (2) intellectual or physical impairments or uncorrectable sensory impairment precluding participation in the laboratory session, (3) known diagnoses of disorders that may interfere with the administration of experimental tests, including schizophrenia, autism, bipolar disorder, or epilepsy (participants were asked whether they were ever diagnosed for these disorders by a physician). We did not make exclusions for other, more common clinical conditions to ensure that the sample is representative of the general population; (4) inability to understand English; and (5) history of head trauma with loss of consciousness for more than 5 min. Participants were screened for these exclusion criteria using a telephone interview. It is important to note that twins were ascertained randomly from the local population based on birth records and, apart from the exclusion criteria specified above, the current sample is broadly representative of the general population. Upon arrival to the lab, they also completed a urine drug test [for Methamphetamine, Opiates, PCP, Benzodiazepines, Methadone, Barbiturates, Amphetamines, Cocaine, and TetraHydroCannabinol (THC)] and an alcohol breathalyzer test. One participant’s session was rescheduled because of a positive drug test for THC. The Human Research Protection Office at the Washington University School of Medicine approved the study. A written informed consent was obtained from all participants. Participants were compensated for participation in the study.

### In-Scanner Stop Signal Task (SST) Description

**FIGURE 1:**
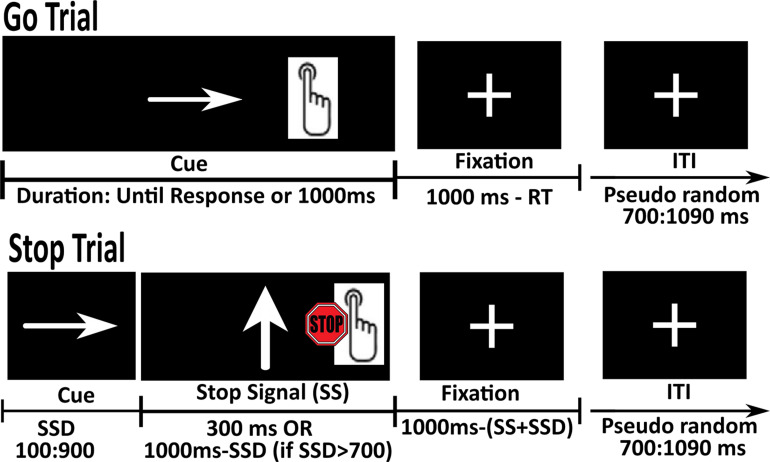
Schematic representation of the Go and Stop trials in the Stop Signal Task (SST). Go trials were composed of the presentation of a left or right pointing arrow (terminated by the response). During Stop trials, subjects were required to withhold their response when the ‘Go’ stimulus was followed by an unpredictable ‘Stop’ signal (an arrow pointing up).

We used a scanner version of the SST developed by Logan (1994) and identical to that administered in the ABCD study (Casey et al., 2018). Before the actual scanning, participants were placed in a mock scanner for accommodation to the scanner environment, where they received instructions and performed a practice version of the in-scanner tasks (for details see Supplementary Materials). In the SST, participants were instructed to press a button that corresponds to the direction of an arrow presented on the screen (press a button with the pointer and middle finger, respectively, for the arrow pointing to the left and right), as quickly and accurately as possible (‘Go’ response). In 1/6 of the trials, they were required to stop or withhold their response to the ‘Go’ stimulus when it was followed by an unpredictable ‘Stop’ signal (an arrow pointing up) (see Figure 1).

The task was administered in 2 runs, 180 trials each (150 Go and 30 Stop trials). Each run started with a fixation period of 2 s. The total duration of each trial was set to 1 s, followed by an Inter Trial Interval (ITI). Go trials were composed of the presentation of a left or right pointing arrow for the duration of 1 s or until response, whichever comes first, followed by presentation of a fixation cross with variable length. Stop trials were composed of the presentation of a left or right pointing arrow for the Stop Signal Delay (SSD) as determined by an algorithm, followed by a Stop Signal for 300 ms and a fixation cross of variable length. If the duration of SSD was greater than 700 ms, the duration of the Stop Signal was calculated as (1 s-SSD). ITI varied between 700 to 1090 ms (note that ABCD used different ITI durations). Participants’ accuracy was tracked during the task and SSD was varied in order to maintain a 50% success in Stop trials. The duration of the SSD was set to 50 ms at the beginning of the task and restricted to remain < 900 ms. Following a correct Stop response (participant successfully inhibited their response), the duration of the SSD was increased by 50 ms to make the task more difficult. Conversely, following an incorrect Stop response (participant not able to inhibit response), the SSD was decreased by 50 ms to make the task easier. This adaptive procedure was implemented to ensure ∼50% accuracy on the Stop trials. Stop trials were interspersed between 3 to 7 Go trials. If the participant gave a motor response before the presentation of the Stop Signal, that trial was counted as a correct Go trial and the Stop Signal was presented in the next trial. Leftward- or rightward-pointing arrows were presented equally often in the Go and Stop trials. Each run ended with a fixation period of 5 s.

The main behavioral outcome variable in this task is Stop Signal reaction time (SSRT) and was calculated based on (Eagle et al., 2008).

During each scanning session, participants performed six cognitive tasks in a predetermined order, with the two 6-min runs of the SST presented as the first task. All subjects completed both runs of the SST at both timepoints.

This task has a number of limitations that could affect the interpretation of behavioral measures as highlighted in a recent report (Bissett et al., 2020), but the extent to which these design features could affect brain activation is not yet clear.

### fMRI Data Acquisition

Echo-planar imaging (EPI) of the whole brain was acquired with a 32-channel head coil on a 3T Siemens MAGNETOM Prisma scanner in the WUSM Neuroimaging Labs, using Human Connectome Project (HCP) style acquisitions. The specific sequence implementations and scanning parameters were identical to those used for the Adolescent Brain Cognitive Development (ABCD) Study (Casey et al., 2018). Structural scans included a sagittal magnetization prepared gradient-echo (MP-RAGE) T1-weighted image (repetition time [TR] = 2500 msec; echo time [TE] = 2.88 msec; flip angle = 8^0^; voxel size = 1.0 mm × 1.0 mm × 1.0 mm) and a sagittal T2-weighted image (T2-SPACE, TR = 3200 msec; TE = 565 msec; voxel-size = 1.0 mm × 1.0 mm × 1.0 mm). Both the T1w and T2w scans utilized embedded volumetric navigators that detected and compensated for head movement in real-time, with an allowance for reacquisition of the lines (TRs) in *k-*space that are heavily corrupted by motion (up to 24 TRs for the MP-RAGE, and 18 TRs for the T2-SPACE scan). The combination of real-time motion correction and *k-*space reacquisition improves the quality of the structural scans and reduces the need for rescans, especially for age groups with a higher incidence of head movement (Tisdall et al., 2012). BOLD contrast for the task was measured with a gradient-echo EPI sequence (TR = 800 msec; TE = 30 msec; 445 frames; 60 contiguous 2.4 mm transverse slices; 2.4 mm × 2.4 mm in plane resolution, multi-band factor 6, posterior-to-anterior phase encoding). Two brief spin-echo EPI scans with opposite phase-encoding directions (anterior-posterior and posterior-anterior) were acquired immediately before the BOLD scan for the purpose of correcting susceptibility distortion.

### fMRI Data Processing

Functional Magnetic Resonance Imaging data processing was identical to that described in Korucuoglu et al. (2020). In short, the HCP data analysis pipelines (^1^ v.3.19.0) were used for the preprocessing of fMRI images (Glasser et al., 2013). The *PreFreeSurfer*, *FreeSurfer*, and *PostFreeSurfer* pipelines were used for structural processing, after which the structural results underwent careful quality control (see Supplementary Materials). The *fMRIVolume* and *fMRISurface* pipelines were used for fMRI preprocessing. Then the *TaskfMRIAnalysis* pipeline (v.4.0.0), which uses FEAT tool (FMRIB’s Expert Analysis Tool) from FSL v6.0 (Jenkinson et al., 2012), was used to analyze the cortical and subcortical grayordinate data for task modeling. The first eight frames were discarded from further analysis to allow for equilibrium of the longitudinal magnetization.

The fMRI model included 4 regressors: Correct Go, Correct Stop, Incorrect Stop, and Other Errors. Other Errors was composed of omission errors (no response to a Go stimulus), incorrect response to a Go stimulus (pressing the button assigned to the arrow pointing the other direction), and late Go responses (responses during ITI). Note that Go trial error types were rare (mean omission errors = 2.11, mean incorrect Go errors = 4.38, mean late Go errors = 5.0), and as a result not all subjects had each error type or a sufficient number in order for each error type to be modeled as a distinct event type. Nevertheless this procedure allowed separation of activation related to Go error types from baseline. At the time of the analysis ABCD SST model predictors included a ‘Failed Go Trials’ regressor (in addition to Correct Go, Correct Stop, and Incorrect Stop regressors as above), however specific error types that are included in this category were not detailed (see ABCD 2.0 Release Notes). All regressors were modeled with a duration of 1 s, from the onset of the stimulus presentation (left and right pointing arrow). We focused on two contrasts: (1) *Correct Stop vs. Correct Go contrast* was used to study neural correlates of successful inhibition; (2) *Incorrect Stop vs. Correct Go* contrast was used to evaluate brain activity related to error monitoring, as it was implemented in the ABCD Study. It is important to note that in the *Incorrect Stop vs. Correct Go* contrast a motor response was present in both events; therefore error-related activity was not confounded with activity associated with a motor response. We did not correct for the multiplicity of contrasts.

### Definition of Brain Regions Analyzed

In our analytical strategy, we strived to balance a data-driven, exploratory approach and an *a priori* hypothesis-driven approach to the identification of reliable neural markers of RI and EM.

First, to provide full and unbiased data, we report reliabilities for the entire brain at the parcel level (referred to as *unthresholded parcels* analysis). Another reason for estimating TRR in regions that may not show a significant task-related activation is the possibility that some regions may show low task-related activity at the group level but at the same time exhibit large inter-individual differences in the strength and even the direction of activation, which can be reliable within an individual. For the *unthresholded parcels* analysis, whole brain grayordinate-wise beta weights were divided into 360 anatomical areas using the Human Connectome Project Multi-Modal Parcelation, version 1.0 (MMP1.0, Glasser et al., 2016). The MMP1.0 parcelation is based on a combination of cortical architecture, function, connectivity, and topography. The beta weights from each parcel were averaged and extracted. Averaging within parcels increases signal-to-noise ratio in each region, reduces data dimensionality and increases statistical power (Glasser et al., 2016). Subcortex areas were based on the FreeSurfer-derived 19 structure subcortical segmentation (Fischl et al., 2002) embedded into the definition of the CIFTI grayordinate standard space by the HCP Pipelines.

Second, we identified brain regions that had *a priori* theoretical importance due to their association with RI and EM based on the meta-analytic studies of Swick et al. (2011) and Neta et al. (2015) (referred to as *selected ROIs*). For *selected ROI* analysis, MMP parcels corresponding to the regions reported in the meta-analytic studies of Swick et al. (2011) and Neta et al. (2015) (Table 2 in the cited article) were identified by using the MNI coordinates for the peak voxels. The *unthresholded* beta weights from each parcel were averaged and extracted, therefore ROIs contained data from all vertices/voxels within that parcel/segment.

Additionally, we provide a summary of our results for the *thresholded parcels* (regions showing significant task-related group level activations in the present study) in the Supplementary Results section. Test-retest reliabilities were estimated for all three sets of data.

### Estimation of Motion

The rotation and translation motion parameters per volume (‘prefiltered_func_data_mcf.par’ output file) were estimated by the HCP *fMRIVolume* pipeline (using FSL’s MCFLIRT tool). The average of the frame-to-frame movement for each run (‘Movement_RelativeRMS_mean.txt’ output file) was calculated for Time1 and Time2, and then averaged across Time1/Time2 for each person.

### Test-Retest Reliability Estimates

Test-retest reliabilities (TRRs) were estimated for the behavioral measures, the *unthresholded* parcel-level data and for the *selected ROIs* before and after the data were cleaned with a noise reduction method, named ICA-FIX (see section, *Data cleaning using multirun ICA-FIX*). In this study, reliability was quantified as the degree of consistency between the Time1 and Time2 measurements, under the assumption of a two-way mixed model, which is known as ICC(3,1) (Shrout and Fleiss, 1979), or alternatively ICC(C,1) (McGraw and Wong, 1996). The relevant mean squares were estimated using method of moments estimators and a Matlab function (‘ICC.m’^2^) based on ICC(C,1) with the use of formulas provided by McGraw and Wong (1996) (referred to as ‘TRR ICC’ in text). Note that this estimator allows for negative ICCs, which were retained in the data to maintain the overall distribution of reliabilities.

Cicchetti (1994) proposed that ICCs are considered poor, fair, good, and excellent with ICC < 0.4, 0.4 < ICC < 0.59, 0.6 < ICC < 0.74, 0.75 < ICC < 1, respectively. The statistical significance of the ICCs was determined with the use of a permutation method (5000 permutations) as in our previous study (Korucuoglu et al., 2020). This method also provided a convenient mechanism to control for the testing of multiple hypotheses (i.e., across all behavioral variables or across all parcels/segments). In this procedure, the Time2 data were randomly permuted – i.e., relabeled as Time2 for a different participant (without regard to twinship) – and ICCs were re-calculated for each of the 5000 permutations. A null distribution was created by selecting the highest ICC (across behavioral measures or parcels/segments) in each permutation. ICC values greater than or equal to the 95th quantile of this null distribution were considered as statistically significant; therefore *p*-value cutoff for all significant ICC values reported in this paper corresponds to ≤ 0.05. Permutations were completed separately for the behavioral variables, *unthresholded parcels* and for *selected ROIs* (for the distribution of permuted ICCs, see Supplementary Figures 1–3). Only parcels with significant ICCs are reported in this paper.

The effect of test-retest interval was studied by comparing the average of long-term TRR (calculated separately for Run1 and Run2 across sessions) with short-term TRR (calculated across two runs within the same session, separately for Time1 and Time2 sessions). In addition, the effect of task duration on the TRR was studied by comparing the TRR calculated on the mean BOLD response magnitudes extracted from Run1 (similarly for Run2) across Time1 and Time2 and the TRR estimated based on the duration of the whole task (the mean BOLD response magnitudes extracted from Run1 and Run2).

### Familial Influences

Intrapair twin correlations were calculated using the same ICC(C,1) estimator as in the TRR estimation (see section, *Test-Retest Reliability Estimates*). This estimate of ‘familiality’ was based on the Time1 data (*n* = 27 pairs), to maximize power because the number of full twin pairs was larger at Time1. The same permutation approach that was utilized for the significance testing of the ICCs (see section, *Test-Retest Reliability Estimates*) was also applied for the significance testing of the twin correlations, except in this case, the sibship assignment of “twin 2” was randomly permuted. Twin correlations greater than or equal to the 95th quantile of permuted null distribution were considered as significant; therefore the *p*-value cutoff for all significant MZ twin correlations reported in this paper corresponds to *p* ≤ 0.05. Permutations were completed separately for the behavioral variables, *unthresholded parcels* and for *selected ROIs* (for the distribution of permuted twin correlations, see Supplementary Figures 1, 5, 6). The relationship between familiality, TRR and potential factors that affect the TRR were investigated with the use of *unthresholded parcels*.

### Data Cleaning Using Multirun ICA-FIX

In order to investigate whether ICA-based artifact removal would improve the TRR of brain activations, ICC estimates of the beta weights for the data preprocessed with and without ICA-FIX were compared. ICA-FIX removes spatially specific temporally structured artifacts (Beckmann and Smith, 2005; Smith et al., 2013; Griffanti et al., 2014; Salimi-Khorshidi et al., 2014). Specifically, we used “multirun” FIX (MR-ICA-FIX) implemented in the HCP Pipelines (v.4.0.0, ‘hcp_fix_multi_run’ script) (Glasser et al., 2018). MR-ICA-FIX concatenates a set of fMRI runs [in this case, 2,487 frames (for a subject with complete data) across 5 task-fMRI scans, including one run of a Balloon Analog Risk Task, 2 runs of a Monetary Incentive Delay task and 2 runs of the Stop Signal Task] therefore providing more data to the spatial ICA, to yield better separation of ‘signal’ and ‘noise’ components. MR-ICA-FIX, as applied in this study, included: (1) demeaning, highpass temporal filtering (sigma = 2000 s), and variance normalization (across space) of individual runs to remove linear trends and prepare the runs for concatenation; (2) concatenation of individual runs, followed by a second pass of variance normalization and estimation of the spatial dimensionality of the concatenated data by comparing the eigenvalue distribution to a Wishart distribution (Glasser et al., 2018); (3) MELODIC independent component analysis (ICA) on the concatenated data, with the previously estimated spatial dimensionality (i.e., number of ICA components), producing component spatial maps and timeseries; (4) classification of these components into signal and noise categories by the FMRIB group’s ICA-based “Xnoiseifier” (FIX) trained ICA component classifier with HCP_hp2000.RData as the “training” (classifier) file; (5) “aggressive” regression of 24 motion parameters (which were also temporal highpass filtered with sigma of 2000 s) out of the data and all ICA components, so that all variance related to the 24 motion parameters was removed from both the fMRI data and ICA time series; (6) “non-aggressive” regression of the ‘noise’ ICA components from the fMRI data (Griffanti et al., 2014), in which all ICA component timeseries (both ‘noise’ and ‘signal’) were simultaneously regressed into the concatenated fMRI data and only the variance uniquely associated with the ‘noise’ components was subsequently removed from the fMRI data (thus preserving any shared variance that is also associated with the ‘signal’ components); (7) splitting the cleaned, concatenated data back into the component individual runs, along with restoring the spatial mean and variance profile of the individual runs. Of note, the MELODIC and FIX classification steps are run on the concatenated volumetric data, but other operations (including demeaning, highpass filtering, variance normalization, and regressions) occur concurrently in the CIFTI grayordinates timeseries data as well. Thus, the end result is individual run, MR-ICA-FIX cleaned CIFTI data, of which the cleaned SST runs were then modeled using the HCP *TaskfMRIAnalysis* pipeline (v. 4.0.0) to generate activation beta values (as detailed above).

### Outlier Detection and Exclusion

Each behavioral variable, mean BOLD response magnitude of the *unthresholded parcels* analysis and motion were analyzed for outliers in R^3^. This procedure was applied to the whole sample, separately on the Time1 (*n* = 56) and Time2 (*n* = 44) data. For the outlier detection procedure only, raw values were converted to Z-scores, and then values greater than three standard deviations from zero were recoded as missing values. This procedure was reiterated 10 times since outlier removal changes the shape of the distribution, thus allowing for the emergence of new outliers. With this exclusion procedure, across the Time1 and Time2 data together, 4.17% of data-points from the behavioral data (for the variables listed in Table 1); 0.98 and 0.49% of data-points from the *unthresholded parcels* analysis in the *Correct Stop vs. Correct Go*, and *Incorrect Stop vs. Correct Go* contrasts, respectively; and 2.00% of data-points from the motion data were replaced with missing values. The outlier exclusion procedure was also applied to the fMRI data (beta weights) cleaned by MR-ICA-FIX (0.46 and 0.64% of data-points from the *unthresholded parcels* mean BOLD data were excluded, respectively, for Time1 and Time2, across two contrasts). Before the estimation of TRRs and familiality, analogous timepoint or twin pair of excluded datapoint was also replaced with missing values.

**TABLE 1:** Summary statistics for the behavioral outcome variables of the SST.

	Paired Samples *T*-Test Results				TRR	Familiality	
Variables	N^*a*^	Time1 (m, SD)	Time2 (m, SD)	*p*	(ICC)	N^*a*^	ICC
N Correct Go	39	294.49 (4.08)	292.82 (5.52)	0.097	0.21	23	0.005
N Correct Stop	42	30.83 (2.16)	30.48 (2.60)	0.172	0.76*G	25	0.395
Correct Go mean RT	40	388.74 (47.32)	385.64 (52.86)	0.620	0.69*G	26	0.637*
Incorrect Stop mean RT	42	445.72 (113.12)	433.09 (103.95)	0.360	0.67*G	25	0.560*
SSD	39	136.45 (67.38)	150.34 (99)	0.196	0.70*G	22	0.582*
SSRT	41	225.04 (71.69)	223.94 (55.89)	0.914	0.50*F	25	0.225

^a^Number of subjects per variable varies due to the outlier detection/exclusion procedure, see methods for details; (m, SD), mean, standard deviation; RT, reaction time; N, number; ms, millisecond; F, fair ICC values (0.4 < ICC < 0.59), G, good ICC values (0.6 < ICC < 0.74), based on (Cicchetti, 1994); *significant test-retest reliability/familiality based on 95% quantile of permutations, including control for multiple comparisons.

## Results

### Performance Results

Mean (SD) percent correct stop responses were 51.30% (3.36) at Time1 (*n* = 56) and 50.98% (4.38) at Time2 (*n* = 44) suggesting that the adaptive procedure was successive in achieving an approximately equal number of successful and unsuccessful inhibition (Stop) trials.

Mean values of behavioral outcome variables measured with the SST did not change significantly over time (Table 1). The main behavioral outcome measure of SSRT showed fair but significant TRR (ICC = 0.5) and poor familiality (*r* = 0.23, n.s.).

### fMRI Results

#### Whole-Brain Activation During RI and EM

Group level activation maps for RI and EM are presented in Supplementary Figure 10.

Supplementary Table 2 lists *a priori selected regions* that are implicated in RI and EM based on meta-analyses conducted, respectively, by Swick and colleagues (Swick et al., 2011) and Neta and colleagues (Neta et al., 2015). Nearly all of the ROIs of RI and EM, identified in the aforementioned meta-analyses, were significantly active in the present study (those parcels/segments showed significant activations either for the whole or part of the vertices within the parcel).

Apart from these *a priori* selected regions, our analysis revealed other regions that were active in both successful and unsuccessful inhibition trials, including but not limited to, the bilateral rostral middle frontal (9_46d), precentral (6a), caudal ACC (a24pr), superior frontal (a32pr, p32pr, 8BM), rostral ACC (p24), caudal middle frontal (8C, 8Av), parsopercularis (6r and FOP4) of the inferior frontal gyrus, posterior cingulate (RSC), lateral OFC (11l, AVI), the anterior part of the insula (MI), precentral (6r, PEF) and parietal regions (AIP, LIPv, 7PC, LIPd, PF, PFm). For both conditions, the spatial extent of these activations in the right hemisphere was greater and included additional parcels overlapping with the rostral ACC (R_a24), pars triangularis and pars opercularis (R_IFJa, R_IFSp, R_IFSa), rostral middle frontal (R_a10p), inferior parietal (R_PGs), precuneus (31pd), right thalamus, and hippocampus. Parcels that were only active during successful inhibition trials (response inhibition) extended to regions of the bilateral pre- and postcentral gyrus (3b, 5mv, 1, 3a, 6d, 6mp), right frontal pole (R_p10p), right posterior part of the insula (R_52, R_RI), rostral middle/lateral orbitofrontal (R_10pp, R_p10p), left superior temporal (L_Pbelt), right posterior regions including fusiform and parahippocampal areas (R_PHA1, R_PHA3), left accumbens and bilateral caudate. Parcels that were only active during failed inhibition trials (error monitoring) included the left thalamus, left hippocampus, R_PeEc (fusiform), L_8BL (superior frontal), L_a47r (rostral middle frontal), L_Op1 (postcentral), L_Pir, L_52, L_RI (insula), and parcels at temporal regions (L_Tgd, L_Te1p, L_Te2a, L_Tgv).

#### Test-Retest Reliability

##### Reliability of inhibition-related activity

**FIGURE 2:**
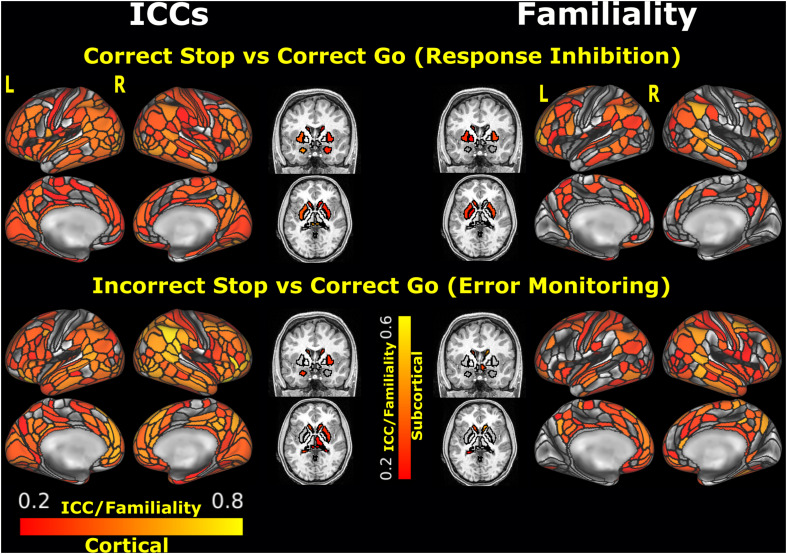
ICC estimates and MZ twin correlations for the *unthresholded parcels* analysis. *Black* outlines depict the boundaries of the MMP1.0 cortical parcelation and the Freesurfer segmentation. Parcels/segments with ICCs < 0.2 are not plotted. L: left, R: right. [ICCs of the *unthresholded parcels* can be found in the BALSA repository for neuroimaging data: https://balsa.wustl.edu/study/show/wN9n4].

Among the *unthresholded parcels*, significant fair to good ICC values (0.52–0.65) were found for the *Correct Stop vs. Correct Go* contrast including the following regions: lateral orbitofrontal (47s), right rostral middle frontal (R_9p, R_IFSp), right caudal middle frontal (R_IFJp), the right posterior cingulate (R_31a), and left insula (L_Pol1) (for a complete list, see Supplementary Table 1 and Figure 2). Among the *selected ROIs* (see Supplementary Table 2), significant ICCs were observed, from highest to lowest, in the left middle occipital gyrus, right middle frontal gyrus, right inferior occipital gyrus, left precentral gyrus, left middle temporal gyrus, right superior parietal gyrus, and left insula, all within the range of fair ICCs (ICCs ranging from 0.43 to 0.61).

##### Reliability of error-related activity

Among the *unthresholded parcels*, significant ICC values ranged from 0.52 to 0.77 for the *Incorrect Stop vs. Correct Go* contrast, including left and right lateral orbitofrontal (L_47s, R_47s), medial orbitofrontal (L_10r, L_p32, R_p32), superior frontal and rostral middle frontal (extending multiple parcels), rostral anterior cingulate (L_d32, L_9m, R_p24, L_a24, L_p24), and right insula (R_AAIC) (for a complete list, see Supplementary Table 1 and Figure 2). Among the *selected ROIs* (see Supplementary Table 2), significant TRR was observed in the left and right anterior insula, medial superior frontal and dorsolateral prefrontal cortex.

#### Familiality and Its Relationship With Test-Retest Reliability

The spatial distribution of the MZ twin correlations at Time1 for the *unthresholded parcels* is presented in the Figure 2. Among all *unthresholded parcels*, only the left superior frontal (L_8BL) showed fair-to-good values (and statistically significant) for both ICC and familiality estimates, in the *Incorrect Stop vs. Correct Go* contrast. Among *selected ROIs*, right superior temporal area (R_STSdp) in the *Correct Stop vs. Correct Go* contrast and right DLPFC (R_55b) in the *Incorrect Stop vs. Correct Go* contrast had statistically significant ICC and familiality estimates.

**FIGURE 3:**
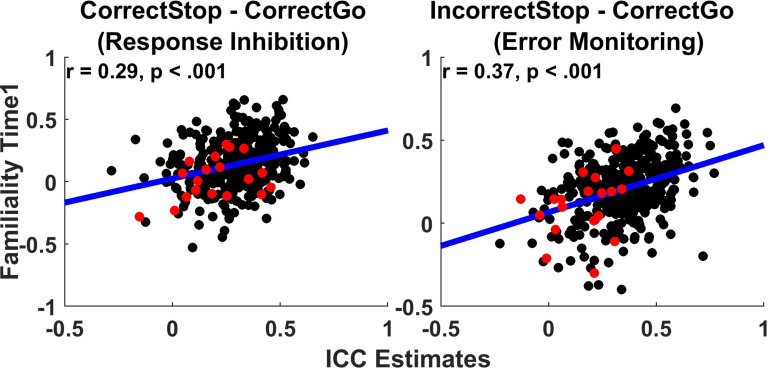
Test-retest reliability weakly correlates with Familiality. Scatterplots display test-retest reliabilities (ICCs) and familiality (MZ twin correlations) for the whole brain cortical MMP parcels and subcortical brain regions (*unthresholded parcels* analysis). Each data-point represents a parcel/segment. Regression lines and correlations are calculated based on the joined cortical and subcortical data. Black: Cortical parcels; Red: Subcortical segments.

Figure 3 shows the relationship between TRR (ICCs) and familiality (MZ twin correlations) at Time1 for the *unthresholded parcels*. Across all parcels, familiality ranged from −0.52 to 0.65 and −0.40 to 0.69 for the *Correct Stop vs. Correct Go* and *Incorrect Stop vs. Correct Go* contrasts, respectively. Weak to moderate positive relationships were found between TRRs and intrapair twin correlations (*r* = 0.29 and 0.37, respectively, for RI and EM-related activations).

Table 2 provides an overall summary of correlations between measures of TRR and familiality (MZ twin correlation), separately for the cortical and subcortical regions. These correlations show the extent to which reliability relates to familiality. Across the cortical parcels, TRR correlated with familiality weakly in the *Correct Stop vs. Correct Go* (*r* = 0.27, *p* < 0.01) and moderately in the *In*c*orrect Stop vs. Correct Go* contrast (*r* = 0.36, *p* < 0.01). In subcortical regions, reliability did not correlate with familiality (all *p* > 0.05).

**TABLE 2:** Correlations between measures of test-retest reliability (TRR ICCs), effect sizes (Cohen’s d), Time1 activation (beta weights, mean and standard deviation across participants), and familiality (MZ twin correlations) for the *unthresholded parcels* analysis (i.e., whole brain parcelation/segmentation).

	Cortical MMP Parcelation						Subcortical Freesurfer Segmentation					
	Response Inhibition											
**Correct Stop vs. Correct Go**		**ICC**	**CohensD**	**MBetas**	**SDBetas**	**Familiality**		**ICC**	**CohensD**	**MBetas**	**SDBetas**	**Familiality**
		
	**ICC**	1	0.26**	0.31**	0.29**	0.27**	**ICC**	1	0.07	0.03	0.49*	0.31
	**CohensD**		1	0.97**	−0.11*	0.11*	**CohensD**		1	0.99*	0.02	0.09
	**MBetas**			1	0.002	0.17**	**MBetas**			1	−0.008	0.05
	**SDBetas**				1	0.32**	**SDBetas**				1	0.50*
	**Familiality**					1	**Familiality**					1

**Error Monitoring**												

**Incorrect Stop vs. Correct Go**		**ICC**	**CohensD**	**MBetas**	**SDBetas**	**Familiality**		**ICC**	**CohensD**	**MBetas**	**SDBetas**	**Familiality**
		
	**ICC**	1	0.35**	0.34**	0.43**	0.36**	**ICC**	1	0.07	0.02	0.05	0.38
	**CohensD**		1	0.98**	0.03	0.09	**CohensD**		1	0.99**	−0.09	−0.01
	**MBetas**			1	0.04	0.08	**MBetas**			1	−0.07	−0.07
	**SDBetas**				1	0.19**	**SDBetas**				1	0.24
	**Familiality**					1	**Familiality**					1

ICC, Intraclass correlation coefficients; CohensD, effect sizes based on Time 1 data (mean/SD of beta weights across subjects -based on full sample except outliers-, calculated per parcel); MBetas, Time 1 beta weights; SDBetas, standard deviation of Time 1 beta weights; Familiality, correlations of beta weights between monozygotic twins. Pearson correlations, *<0.05, **<0.01.

#### Factors Potentially Affecting TRR

##### How does retest interval affect TRR?

To address this question we computed a paired samples *t*-test comparison of r-to-z transformed ICC values between ‘within-session ICCs’ (reliability computed across first and second run of the task, separately per timepoint, then averaged across timepoints) and long-term reliability estimates (between-session ICCs, calculated per run separately, then averaged across runs). Both for RI- and EM-related activations, within-session ICCs (mean ICCs = 0.22 and 0.33, respectively for RI and EM) were greater compared to between-session ICCs (mean ICCs = 0.19 and 0.27, respectively for the RI and EM) (*t* = −5.49 and −9.38, respectively for the RI and EM, all *p*s < 0.001).

##### Does within-session reliability relate to long-term reliability?

Correlations between ‘within-session ICCs’ and long-term reliability estimates (between-session ICCs) are presented in the Supplementary Figure 7. Overall, within- and between-session ICCs demonstrated weak but significant correlations, suggesting that within-session reliability is a moderately strong predictor of long-term, between-session reliability.

##### Does TRR depend on task/scanning duration?

To address this question, we computed a paired samples *t*-test comparison of r-to-z transformed ICC values between long-term ICCs computed for beta weights extracted from single runs (6 min) with ICC computed using both runs (12 min, ICCs calculated based on beta weights extracted from per subject averaged parameter estimates, a.k.a. 2nd level analysis). Both for RI and RM-related activations, single run ICCs (both Run1 and Run2) were lower than ICCs computed using both runs (all *p*s < 0.001). Moreover, ICCs computed using Run1 were also greater than ICCs computed using Run2 (all *p*s < 0.001), suggesting slightly greater reliabilities for the first run of the task. Average ICC values across all parcels for RI- and EM-related activations were 0.21 and 0.31, respectively, for Run1, 0.17 and 0.26, respectively, for Run2, and 0.30 and 0.36, respectively, for both runs combined.

**FIGURE 4:**
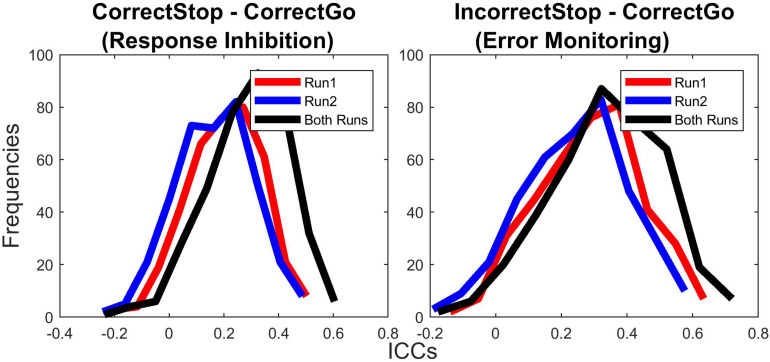
Test-retest reliability was greater for longer task duration. Test-retest reliabilities (ICCs) of unthresholded parcels for both runs were greater than for single run (run1 and run2).

Figure 4 shows the distributions of ICCs across 379 cortical and subcortical parcels separately for Run1, Run2, and full (Run1 + Run2) data. Figure 4 reveals that pooling data from both runs at each time point (session) does lead to an overall increase in ICCs for both RI- or EM-related activations, suggesting that, increasing task duration from 6 to 12 min provide a gain in TRR.

##### Is the magnitude of activation associated with test-retest reliability?

To address this question, we computed correlations across parcels/segments between magnitude (mean beta) and the effect size (Cohen’s d) of unthresholded activation within a parcel with the TRR of the mean beta weights within that parcel (Table 2). Across the cortical parcels, effect size and magnitude of the Time1 activations showed moderate positive correlations with the TRR for the RI-related (*Correct Stop vs. Correct Go)* and the EM-related (*Incorrect Stop vs. Correct Go)* contrasts (*r* = 0.31 and 0.34, respectively), suggesting that activation magnitude moderately related to TRR. This relationship was observed for cortical parcels only and was non-significant for subcortical segments.

##### How does in-scanner motion affect intra-individual stability of brain activation?

**FIGURE 5:**
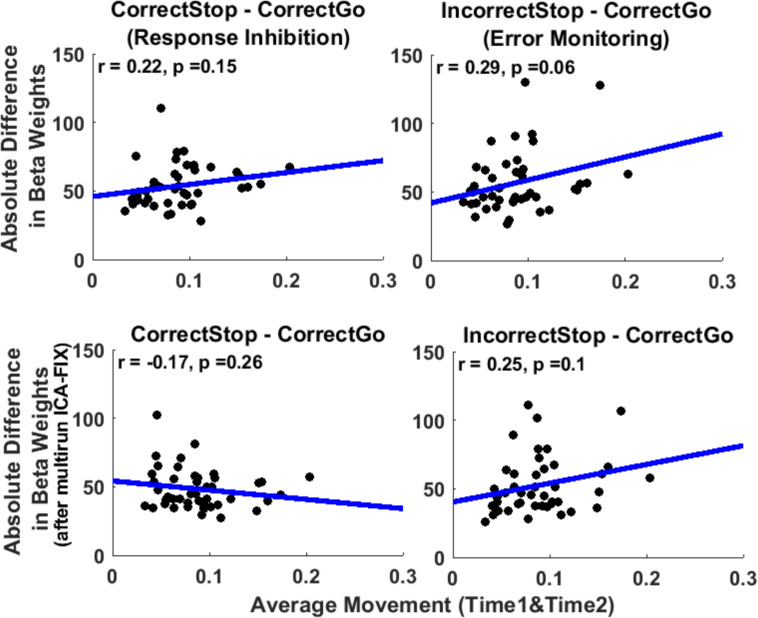
In-scanner movement correlates weakly with intra-individual variability of activation averaged across whole parcels across sessions. However, this relationship disappeared after cleaning the data with multirun ICA-FIX. Scatterplots of average movement (across run and Time1 and Time2) and disparity in beta weights (absolute difference across Time1 and Time2, from the *unthresholded parcels* analysis) (**upper** panel). The same correlations after cleaning the data with multirun ICA-FIX (**bottom** panel). Each data-point represents a participant’s data averaged across parcels. Blue line: regression line. Units of movement (mm).

Since test-retest reliability (ICCs) is a group level measure, it is not possible to correlate ICC with participant-level motion. Instead, we examined the relationship between the average amount of motion across the two sessions (Time1 and Time2) for each participant and absolute difference in beta weights from Time1 to Time2 for each participant averaged over all *unthresholded parcels* (Figure 5). We expected that individuals with greater motion would tend to show larger absolute inter-session differences in activation indicating lower intra-individual stability. Consistent with this hypothesis, analysis revealed a trend level weak correlation between motion and disparity of regional activation across sessions for EM-related activation (*r* = 0.29, *p* = 0.06), suggesting that a greater amount of in-scanner motion is associated with larger within-subject variability from session to session, which should decrease TRR. However, for RI the correlation was non-significant, albeit in the expected direction (*r* = 0.22, *p* = 0.15).

##### Does ICA-based artifact removal improve the test-retest reliability of brain activations? (unthresholded parcels and selected ROIs)

**FIGURE 6:**
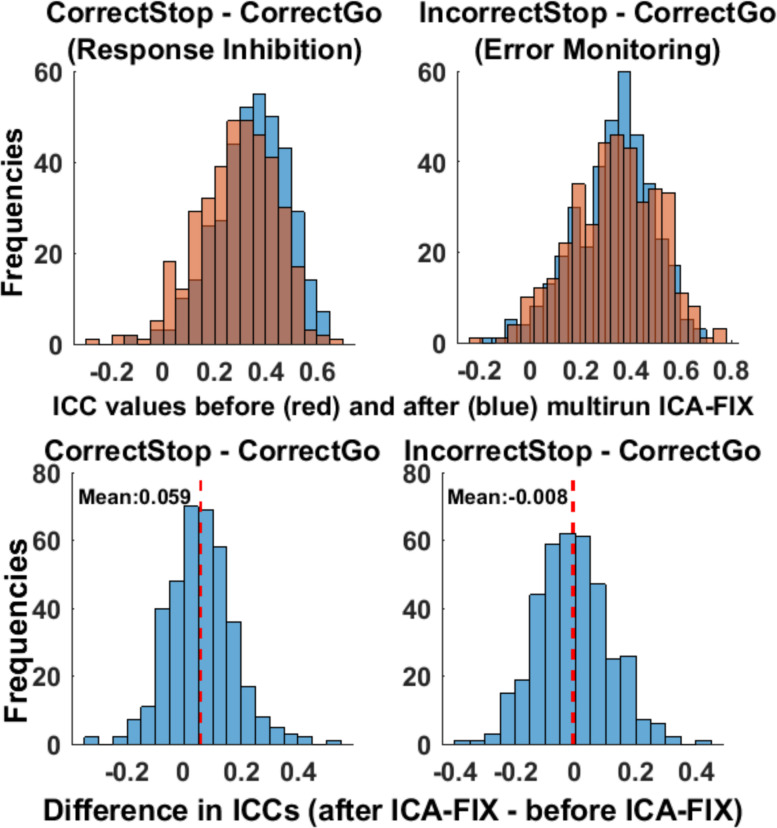
ICC estimates slightly improved after cleaning the data with multirun ICA-FIX. The distribution of ICCs before and after cleaning the data with multirun ICA-FIX, for the unthresholded parcels/segments (**upper** panel) and histogram of differences in ICCs before and after cleaning the data with multirun ICA-FIX (**lower** panel). Each data-point represents an *unthresholded parcel/segment*.

Figure 6 depicts the distribution of ICC estimates across the 379 parcels/segments that cover the entire brain (*unthresholded parcels*) before and after cleaning the data with ICA-FIX. ICC estimates were slightly greater after cleaning the data with multirun ICA-FIX for the *Correct Stop vs. Correct Go* contrast (average difference = 0.06, *unthresholded parcels*, paired *t*-test *p* < 0.001). As presented above, motion was one of the factors that moderately affected the ICC estimates. Therefore, we looked at whether the correlation between the stability of beta weights and motion remained after ICA-FIX cleaning (see Figure 5, bottom panel). As can be seen, that correlation disappeared after cleaning, suggesting that multirun ICA-FIX might improve reliabilities, at least partially by removing structured artifacts related to motion from the fMRI data.

ICC estimates of *selected ROIs* from the data cleaned with the multirun ICA-FIX method can be found in Supplementary Table 2. Among the *selected ROIs*, the effect of multirun ICA-FIX precleaning was contrast dependent. For the *Correct Stop vs. Correct Go* contrast (response inhibition), the overall TRRs were greater for the data with cleaning compared to the data without (with an increase in ICC estimates of 0.08). For the *Incorrect Stop vs. Correct Go* contrast (error monitoring), the results were mixed: While some regions showed an increase, others showed a decrease in ICCs after cleaning.

## Discussion

The aims of this study were to estimate TRR and familiality of individual differences in behavioral and neural correlates of RI and EM, to identify brain regions with high reliability and familiality that can be used as neural markers or intermediate phenotypes (endophenotypes) in clinical and genetic studies, and to examine the factors potentially affecting TRR.

Test-retest reliability of *brain activation* measures was low overall but varied as a function of a specific condition (contrast) and brain region. For successful inhibition trials, we found good reliabilities (some of them only after cleaning the data) in regions consistently implicated in response inhibition by the previous literature – the inferior frontal, middle frontal gyrus, superior parietal and precentral gyrus. Brain activity associated with error responses (unsuccessful inhibition) showed fair to good reliabilities in regions implicated in error monitoring, including the insula, medial superior frontal, and dorsolateral prefrontal areas.

### Test-Retest Reliabilities of Behavioral Measures

Stop Signal Reaction Time (SSRT), the main behavioral measure in the SST that quantifies the latency of inhibition, showed fair reliability (ICC = 0.50), though that is still in the range of previous reports. Wöstmann et al. (2013) reported an ICC of 0.03 for the SSRT in a test-retest study with an 11 weeks interval (*n* = 23). Congdon et al. (2012) reported split half ICC of 0.71 for SSRT in a much larger sample (*n* = 129), with lenient outlier criteria and using the average of 2 runs. Kindlon’s study (Kindlon et al., 1995) reported test-retest correlations of 0.66 in SSRT, which decreased to 0.42 when age effects were controlled for. SSRT is a complex measure that is indirectly modeled with the use of Go reaction time and Stop Signal Delay (SSD), which might be affected by different processes in the task. This complexity may explain its relatively modest reliability.

### Test-Retest Reliabilities of Neural Activity During Response Inhibition (Successful Stop Events)

A published meta-analysis focusing on inhibitory control processes (successful inhibition trials) found that activation in the right IFG but not the right insula predicted individual differences in response inhibition (Cai et al., 2014; for similar results in a Simon task, see Forstmann et al., 2008). Our findings suggest that inhibition-related activations clustered around these regions might be reliable but not susceptible to familial influences. However, we identified one ROI at the right superior temporal area (R_STSdp) with significant fair reliability and familiality, suggesting that this region might be a reliable endophenotype for use in future genetic studies.

Overall, unthresholded parcels had greater reliabilities than the ROIs that were selected based on previous literature, suggesting that future individual differences studies that utilize SST in general or the ABCD dataset in specific, may benefit more from focusing on unthresholded data with fair reliabilities identified in the present study, such as lateral orbitofrontal, superior frontal, caudal middle frontal, insula and superior parietal regions.

### Test-Retest Reliabilities of Neural Activity During Error Monitoring (Unsuccessful Stop Events)

Among the *selected ROIs*, the bilateral insula showed fair reliabilities and moderate but non-significant familiality for the failed inhibitions. In addition, parcels overlapping with the right medial superior frontal ROIs also showed fair reliabilities, but non-significant poor-to-fair familial effects. Besides two regions that met the joint criteria for genetic studies (reliability and familiality), one ROI at the right DLPFC (R_55b) and one unthresholded parcel at the superior frontal region (L_8BL), all other regions were unsuitable to be utilized as neural markers in future clinical and genetic studies.

In an attempt to differentiate the role of the anterior insula and IFG, Cai et al. (2014) conducted a meta-analysis and reported that while both regions showed similar activation levels during successful Stop vs. Go trials, the anterior insula revealed greater activation during unsuccessful Stop trials (errors). In light of evidence showing anterior insula involvement in stimulus saliency (Menon and Uddin, 2010) and detection of infrequent and unexpected events (Sridharan et al., 2008), the authors concluded that increased insula activity in the unsuccessful Stop trials might be due to the late and unexpected Stop errors. In the current investigation, contrary to successful Stop events, the insula had fair reliabilities during failed inhibitions (note that cleaning the data with ICA-FIX slightly decreased this reliability). Our data showed that TRRs of activations involving the bilateral insula were greater during EM, than RI (see ICCs for bilateral insula ROIs at Supplementary Table 2). However, among the unthresholded parcels, there were several regions with fair-to-good reliabilities and fair (but insignificant) familial effects. These regions included the superior, rostral, and middle frontal areas, lateral orbitofrontal, precentral, parietal areas and precuneus.

### Factors Affecting Test-Retest Reliability

In the current study, we investigated the influence of five factors on TRR: the time interval between measurements (short- vs. long-term reliabilities), scan duration, activation magnitude, motion, and ICA-FIX cleaning on reliability estimates.

The influence of time interval between measurements on TRR was significant, with most measures of regional activation showing larger within-session reliability than between-session reliability. It is important to mention that within-session reliability in the present study (the correlation between two consecutive runs of the task), given the very short (∼2 min) interval between the runs, may also be interpreted as internal consistency reliability or split-half reliability, rather than test-retest reliability. Overall, within-session ICCs were higher than between-session ICCs for the unthresholded task data for both contrasts. Our findings are consistent with a previous reliability study by Bennett and Miller (2013) that also found diminishing TRR with increasing retest interval (20 min vs. 6 months). Overall, correlations between short- and long-term reliabilities were weak, suggesting that within-session reliability may not be a strong predictor of long-term reliabilities. It is also important to acknowledge that within-session reliabilities may be inflated by the contribution of state variance to inter-individual differences such as amount of sleep, mood, etc.

In agreement with our expectation, longer task and scanning duration (two runs vs. one run) increased TRR. This finding is in line with the resting-state connectivity fMRI literature showing a strong dependency of TRR on scanning duration (e.g., Birn et al., 2013). An interesting finding was that reliabilities based on the first half of the task were slightly greater than reliabilities based on the second half, suggesting that gain in reliability, although significant, may be diminishing with increasing length of task. Why is there a decrease in reliability in the second run? A possible explanation is that any increase in reliability due to greater amount of data may be countered by factors that tend to decrease reliability, such as systematic changes in neural activity over the course of the task due to practice effects and greater automation of performance, fatigue, decreasing attention, etc. These factors may decrease within-subject stability of regional brain activation over the course of the task and thus diminish the advantage provided by increasing task duration. For example, it is possible that brain regions involved in cognitive control show stronger activation at the beginning of the task than in its later phases when performance becomes more automated and the need for cognitive control is declining. Currently, standard approaches to task-fMRI analyses are based on the implicit assumption that task-related activations are homogenous over the duration of the task. A systematic research is needed to test whether this assumption is true for cognitive tasks that are most frequently used in task-fMRI research. Furthermore, the dependency of TRR on task duration may be task-specific, which underscores the importance of establishing an optimal duration for individual tasks to maximize data reliability, particularly when planning large scale studies.

Consistent with our hypothesis, regions with greater task-related activation at the group level showed higher reliability, although this relationship was modest, with regional activation magnitude accounting for up to 11% of variance in ICC values across brain regions. One possible explanation is that, despite the general positive association, there are regions with high task-related activation but low reliability and, conversely, regions with weak activation but relatively high reliability. For example, during successful inhibition events, two adjacent parcels, one in the right lateral orbitofrontal (R_AVI) and the other in the right medial orbitofrontal (r_10v) regions with high and low activation, respectively, showed poor (ICC = 0.24) and fair (ICC = 0.51) reliabilities, respectively, i.e., the region with lower task-related activation (R_AVI) showed higher TRR than the region with higher activation (R_10v). It is possible that regions with high activation but low reliability show a positive “obligatory” task-related activation in all or most subjects with little inter-individual variance in the magnitude of such activation. In contrast, regions with low activation but high reliability may show large differences in the strength and even the direction of activation across individuals, such that they show strong activation in some individuals but weak activation or even a de-activation in others. If these individual differences are stable over time and functionally meaningful, then such regions may have potential value as neural phenotypes or biomarkers for genetic and clinical studies, despite the lack of a significant task-related activation at the group level.

Motion in the scanner was associated with lower within-subject stability, such that individuals with larger amount of motion tended to show larger absolute between-session differences in activation magnitude. Although this effect was relatively small, accounting for less than 10% of within-subject variability, this finding suggests that motion is a factor negatively affecting TRR of task-fMRI data. The present findings are in line with our study using a different (risk-taking) task (Korucuoglu et al., 2020). Although the effect of motion was relatively modest in the present sample of young adults, it may be more pronounced in studies of children or individuals with psychiatric disorders such as ADHD who may display substantially more movement in the scanner. Herting et al. (2018) stated that while children tend to move more in the scanner, with maturation they show less motion, which will lead to higher quality data as they age, increasing the reliability of brain activation measures.

A data-driven noise removal method (multirun ICA-FIX) improved the reliability estimates, abolished the effect of in-scanner motion on within-subject test-retest stability, and resulted in an average ∼14% increase in tSNRs (see Supplementary Figure 9). Since the degree of motion within the scanner itself is a reliable trait (Engelhardt et al., 2017), it was not clear *a priori* whether more advanced data cleaning methods that may diminish the effects of motion would increase or decrease TRR. Our findings suggest that, at least in the age period studied here, advanced noise reduction methods can improve the true estimate of activation and thus improve reliability of task-fMRI data. Therefore, it can be recommended that multirun ICA-FIX be used in cleaning fMRI data from developmental samples such as ABCD where substantial amounts of motion can be expected. Another benefit of using multirun ICA-FIX would be that by increasing the reliability of the fMRI signal, it would ease the demand to collect more data for studies in specific populations. These benefits might be even more pronounced for datasets in which greater amount of motion is expected, a question that should be addressed in future studies. However, we acknowledge that this method is not the only one to decrease the impact of motion on reliabilities (Shirer et al., 2015; Dipasquale et al., 2017; De Blasi et al., 2020; Kassinopoulos and Mitsis, 2020). Lund et al. (2005), for instance, reported reduced intra-subject as well as inter-subject variance in BOLD response with the inclusion of motion parameters in the analysis. Application of such methods in cleaning the data can have significant advantages both by increasing the test-retest reliability, but also decreasing the need for longer scans when scanning difficult subject populations.

### Limitations

We acknowledge that given the lack of dizygotic twin pairs in our study, we cannot distinguish between genetic and shared environmental influences in our estimate of ‘familiality.’ However, this measure provides preliminary information about the possibility of genetic influences on certain measures of regional brain activation. Another concern is that the dependencies in the data introduced due to the MZ twins could potentially bias the ICC estimates, since the ICC model did not concurrently model the sibling relationships. In order to investigate this possibility, we re-estimated reliabilities by assigning Twin 1 and Twin 2 of our twin pairs to separate groups, which resulted in two samples with no dependencies (unrelated individuals, see Supplementary Materials). The ICC values averaged over the two independent samples were very similar to those derived using the full sample, with a regression line nearly indistinguishable from the line of identity, indicating that there was no evidence that the MZ twins biased the ICC estimates in any systematic fashion (Supplementary Figure 8). Moreover, besides main effects of specific factors, the interaction between them can also increase and decrease reliability estimates. Teasing apart the influence of interaction between these factors would require multivariate analysis methods with larger sample sizes, which was not feasible to test with the current sample. Lastly, adult task-based fMRI TRRs reported in the current manuscript may not generalize to studies with younger or older sample characteristics. There exists some evidence that BOLD signal reliability in older samples is comparable to findings in young adults, at least for resting state data (Gou et al., 2012; Song et al., 2012). However, low reliability observed in younger samples may be difficult to interpret as this may either be due to true reliability estimates of the BOLD signal itself or true developmental change (Herting et al., 2018), therefore estimating task-based fMRI reliabilities at first in young adults appear to be a good approach.

Recently, some limitations of the ABCD-like SST task used here have been reported that potentially can affect task performance, such as variable duration of the stimuli (Bissett et al., 2020). It is important to note that a broad variety of task designs have been used to study response inhibition, including both behavioral performance and brain activity. At this time, there is currently no universally accepted “golden standard” SST task, primarily because each particular design has its strengths and weaknesses and improving a certain aspect of one task design may lead to a limitation in other aspects, resulting in a trade-off between task features. Some of the limitations identified in the ABCD task may bias some performance measures, hindering direct comparisons with studies using other versions of SST; however, they are unlikely to have a substantial effect on brain activation. The brain activation pattern in the current task is highly consistent with activations reported in previous studies using other SST design (Rubia et al., 2000, 2003; Rubia, 2002) and a meta-analysis of such studies (Swick et al., 2011; Neta et al., 2015), suggesting that the current task works very well as a tool for engaging inhibitory control-related brain regions. Furthermore, any biases in the assessment of brain activation at the group level produced by certain design features are unlikely to have a significant effect on inter- and intra-individual differences and test-retest reliability, as long as all individuals are administered exactly the same version of the task at all testing occasions. Nevertheless, the results of the study, particularly those pertaining to behavioral measures, should be interpreted with caution. The ABCD Study team is currently evaluating the extent to which certain task features might affect performance and brain activation, and we advise the reader to follow this discussion (Garavan et al., 2020).

## Conclusion

As fMRI is being increasingly used to asses individual differences in task-related brain activation, establishing reliability of activation in specific fMRI tasks becomes an essential prerequisite for upholding rigor, reliability, and replicability of cognitive neuroscience research. In the present study, reliability of fMRI-measured task-related brain activation was generally poor, consistent with previous research. However, several regions showed at least fair reliability, including inhibition-related activations clustering around the inferior to middle frontal gyrus and error-related activations in the bilateral insula, superior frontal gyrus, and rostral ACC. Some regions showed both significant reliability and familiality, including the right superior temporal ROI during response inhibition and right DLPFC ROI together with the left superior frontal area during error monitoring. These regions can potentially be useful as endophenotypes for future genetic studies.

Short-term (within-session) reliability was generally higher than long-term (between-session) reliability. The former was poorly associated with the latter, suggesting that reliable, trait-like activation measures for individual differences studies cannot be identified based on short-term reliability alone. The magnitude of activation was related to TRR, but this relationship was modest, with some regions showing high activation but low reliability, and vice versa, suggesting that selecting ROIs based on high task-related activation may not guarantee reliability. Longer task duration increased reliability; however reliability decreased from the first half of the task to the second half, suggesting a diminishing gain in TRR due to the increase in task duration. Lastly, motion reduced reliability but this effect was abolished by the application of the ICA-FIX cleaning.

## Data Availability Statement

The data analyzed in this study is subject to the following licenses/restrictions: Data from this ongoing project can be made available by request to the principal investigator (AA) after de-identification and documentation is completed. Upon the completion of the study, the data will be shared with the scientific community, pending availability of appropriate resources. All code can be supplied upon request and can be freely shared or reused. This is in compliance with the funding body and institutional review board. Requests to access these datasets should be directed to AA, andrey@wustl.edu.

## Ethics Statement

The studies involving human participants were reviewed and approved by the Human Research Protection Office at the Washington University School of Medicine. The patients/participants provided their written informed consent to participate in this study.

## Author Contributions

AA conceived and directed the project. OK and AA designed the experiment. OK, SG, SA, and JK implemented the experiment and supervised imaging and behavioral data collection. OK led the data analyses, prepared the visualization of the data, and drafted the manuscript. MH, AA, DB, JK, SG, and SA contributed substantially to the analysis and interpretation of data and critical revisions of the manuscript for important intellectual content. All authors provided a final approval for the version to be published.

## Conflict of Interest

The authors declare that the research was conducted in the absence of any commercial or financial relationships that could be construed as a potential conflict of interest.

## Footnotes

1 https://github.com/Washington-University/HCPpipelines

2 https://www.mathworks.com/matlabcentral/fileexchange/22099-intraclass-correlation-coefficient-icc

3 https://www.R-project.org/
