# Supplementary material for: Test-Retest Reliability of Neural Correlates of Response Inhibition and Error Monitoring: An fMRI Study of a Stop-Signal Task
Source: Front Neurosci. 2021 Jan 28;15:624911. doi: 10.3389/fnins.2021.624911 (PMC7875883; doi:10.3389/fnins.2021.624911)
Supplement: Supplementary file 1 [file Table_1.doc]

**SUPPLEMENTARY Materials**

**Practice version of SST:**

Participants, who are naïve to experimental protocols, may have a greater level of difficulty in following experimental instructions. Therefore, a practice version of the SST was designed and administered in a mock scanner. Administering this practice in the mock scanner should also alleviate any novelty and anxiety effects, which have been found to compromise the stability of fMRI data (Chapman et al., 2010). The mock practice version of the SST was the same as the in-scanner version of the task, except it consisted of 25 trials (Stop signal presented in 4 trials).

All 56 participants completed the mock practice version of the SST at Time1. Among 44 participants at Time2, 39 of them completed the practice version of the SST (due to scheduling limitations). Before commencement of the task during the real scan session, participants were reminded of the task instructions at both Time1 and Time2.

**Quality Control**

All data underwent a careful quality control (QC). Following the structural pipelines, we examined a number of outputs to verify successful structural processing with the use of the Connectome Workbench software (WB). Using the CIFTI data format, Connectome WB provides flexibility for a variety of visualizations, allowing the user to visualize data jointly on surfaces and volume slices, and see contours outlining the boundaries of cortical and subcortical areas and foci that represent centers of various ROIs projected to the surface (Glasser et al., 2016; Marcus et al., 2011). In structural scans, we checked for errors in white and gray matter boundaries, eye movement artifacts affecting cortical matter, head movement artifacts such as “banding” or blurriness, non-brain tissue included within the pial surface, parcellation and surface labeling errors, and volume and areal registration distortions. For BOLD data, the temporal SNR, motion (both relative, i.e. relative to preceding time point, and absolute), frame-to-frame intensity variation, and spatial smoothness and coverage were checked. Partial coverage loss was an exclusion criterion from the group level permutation statistics; however none of the subjects had partial coverage loss at Time1. More generally, no participants needed to be discarded from the dataset as part of the QC review. All analyses were run on the computer cluster of the Washington University Center for High Performance Computing.

ABCD has described poor performance in the SST as indicated by:

Go trials having (1) <150 GO trials, (2) Correct GO trial percentage <60%, (3) Incorrect GO trial percentage >30%, (4) Late GO trial percentage (summing correct and incorrect trials) >30%, (5) No Response GO trial percentage >30%; and Stop trials having (1) < 30 STOP trials, (2) STOP trial accuracy (i.e., successful inhibitions) <20% or >80% (see ABCD 2.0 Release Notes). In the current sample only one subject had <60% for correct go trials at Time1, none of the other subjects had poor performance based on any of the other ABCD criteria. Note that outlier detection procedure (detailed in the main manuscript) replaced this subject's Go trial behavioral outcome variables with missing values.

**Figure S1.** Permutation distribution of ICCs and MZ twin correlations from the *behavioral* data.


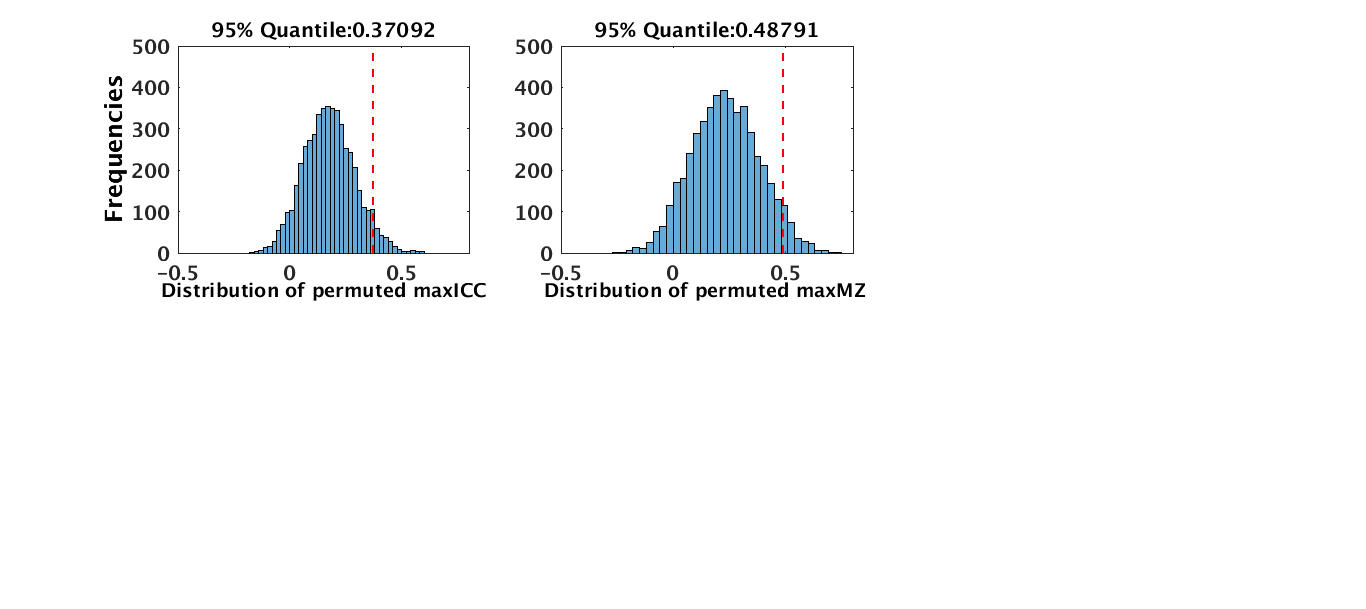


***Notes***. Permuted maxICC/Permuted twin correlations (max). Permutation was used to determine the cutoff for a statistically significant (95th percentile) TRR ICC (left) and twin correlation (right) while also simultaneously controlling for the multiple hypothesis testing of all behavioral variables (6 variables listed in Table1). Each data-point in the distribution represents the maximum ICC/maximum twin correlation across all behavioral variables within a given permutation (5000 permutations total). **Red** dashed line represents the 95% Quantile of this distribution. The Time2 data were randomly permuted (across participants) for the ICC permutations. The Twin2 label of twin pairs was permuted for computing the distribution of the permuted twin correlations, which was computed using the Time 1 data.

**Figure S2.** Distribution of maximum ICC across permutations from the *unthresholded parcels* analysis.

**
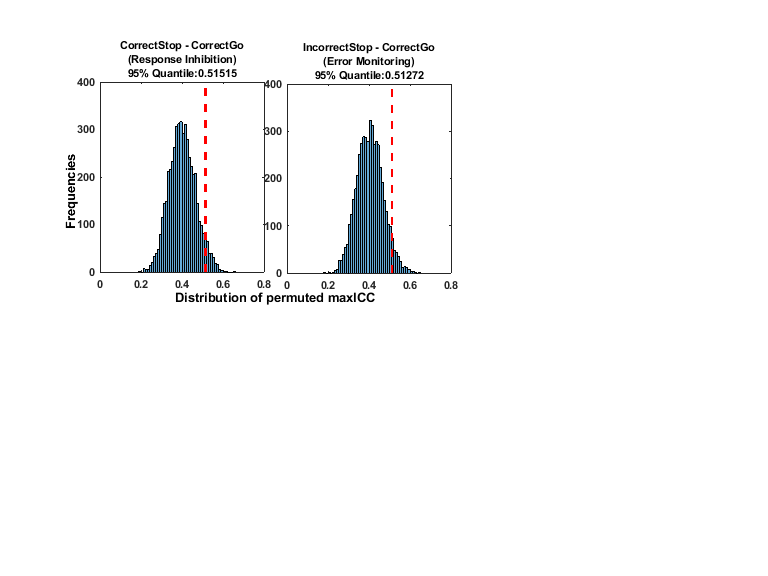
**

***Notes***. Permuted maxICC. Permutation was used to determine the cutoff for a statistically significant (95th percentile) ICC while also simultaneously controlling for the multiple comparisons inherent to the parcellation scheme. Each data-point in the distribution represents the maximum ICC across parcels/segments within a given permutation (5000 permutations total). **Red** dashed line represents the 95% Quantile of this distribution.

**Figure S3.** Distribution of maximum ICC across permutations from the *selected* *ROI* analysis


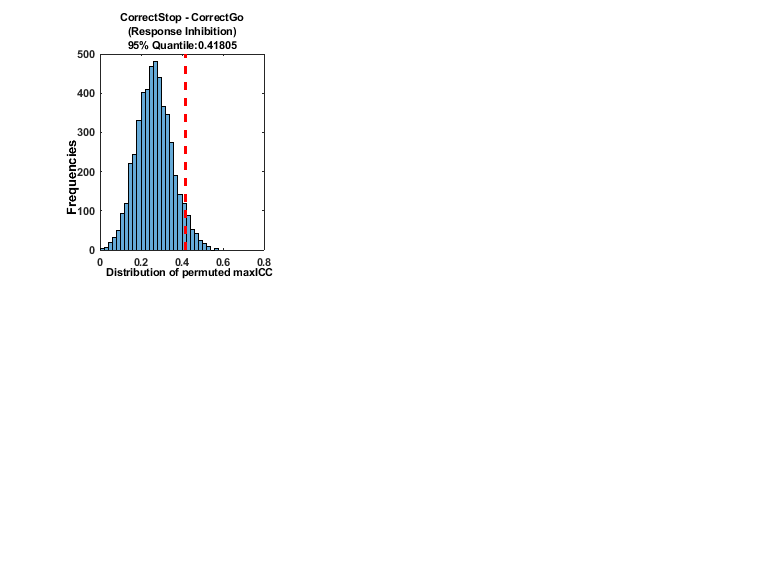

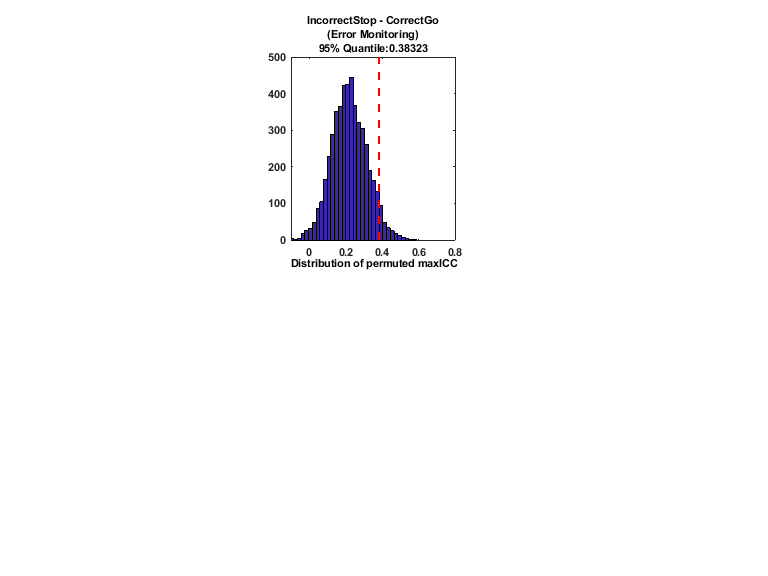


***Notes***. For details, see Figure S2 notes.

**Figure S4.** Distribution of maximum ICC across permutations from the *selected* *ROI* analysis, after cleaning the data with ICAFIX.


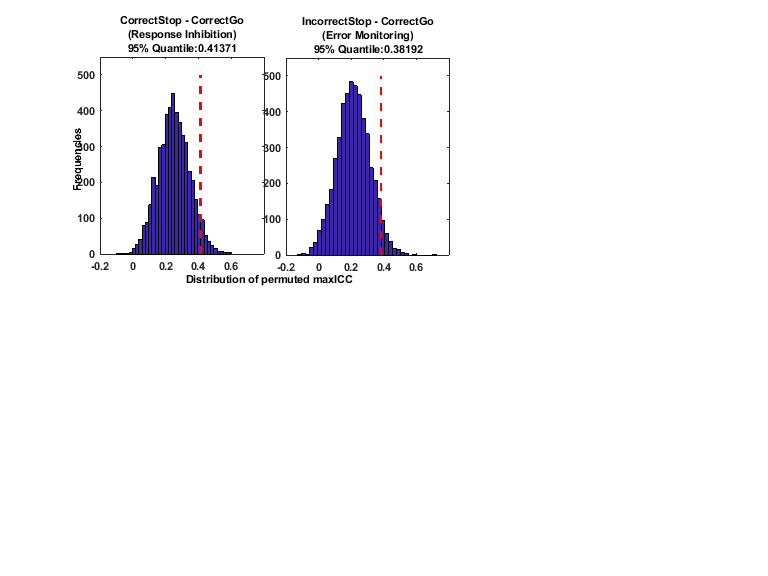


***Notes***. For details, see Figure S2 notes.

**Figure S5.** Distribution of permuted twin correlations from the *unthresholded parcels* analysis.

**
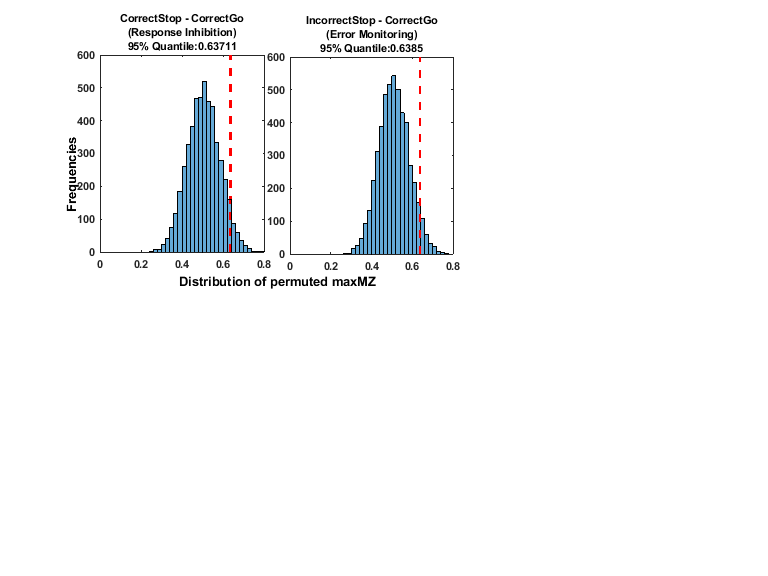
**

***Notes***. Permuted twin correlations (max): each data-point in the distribution represents the maximum twin correlation across parcels/segments within each permutation. The Twin2 label of twin pairs was permuted (5000 permutations). **Red** dashed line represents the 95% Quantile. Twin correlations were calculated from the Time 1 data using the same ICC(C, 1) formula used to compute test-retest reliability.

**Figure S6.** Distribution of permuted twin correlations from the *selected* *ROI* analysis.

**
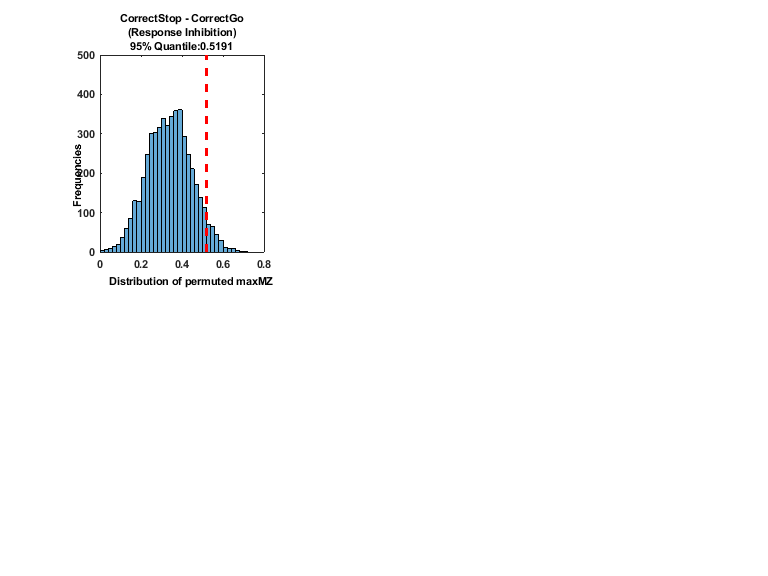
** **
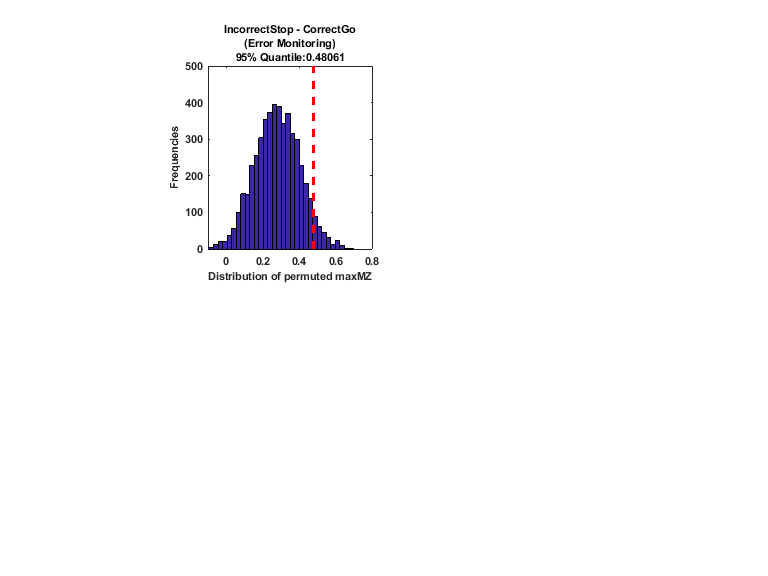
**

***Notes*** For details, see figure S5 notes.

**Figure S7.** Correlations between within-session ICCs (consistency across first and second run of the task, separately for each session -Time1 and Time2 data-) and between-session ICCs (long-term reliability estimates) calculated separately for each of the two 6-min consecutive runs (R1 and R2), as well as ICCs calculated for the full data (R1+R2, ICCs calculated based on beta weights extracted from per subject averaged parameter estimates, a.k.a. 2^nd^ level analysis).

**
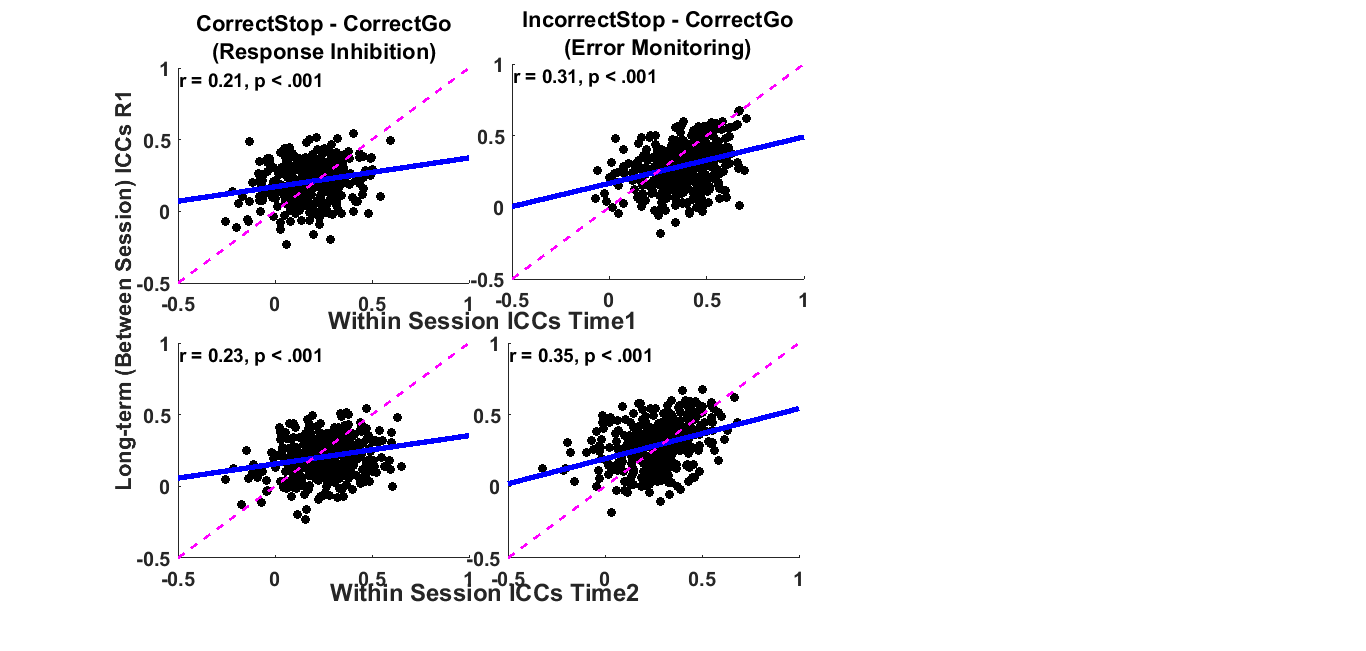
**

**
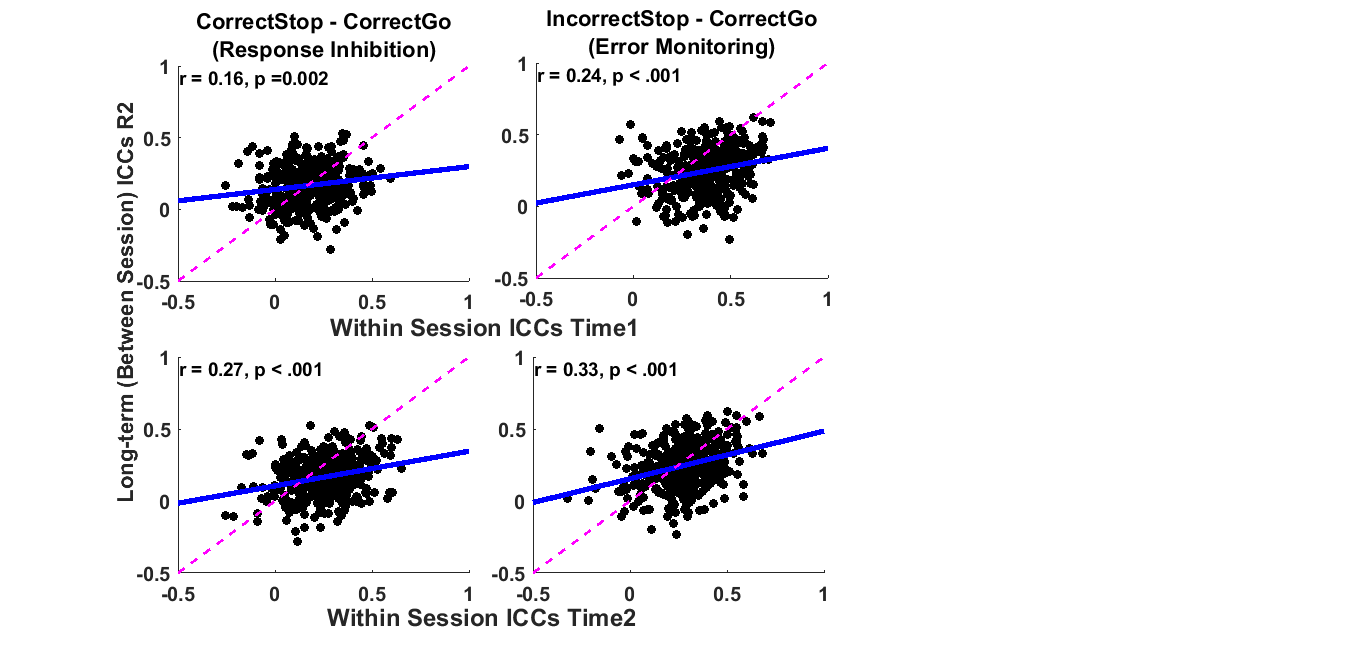
**

**
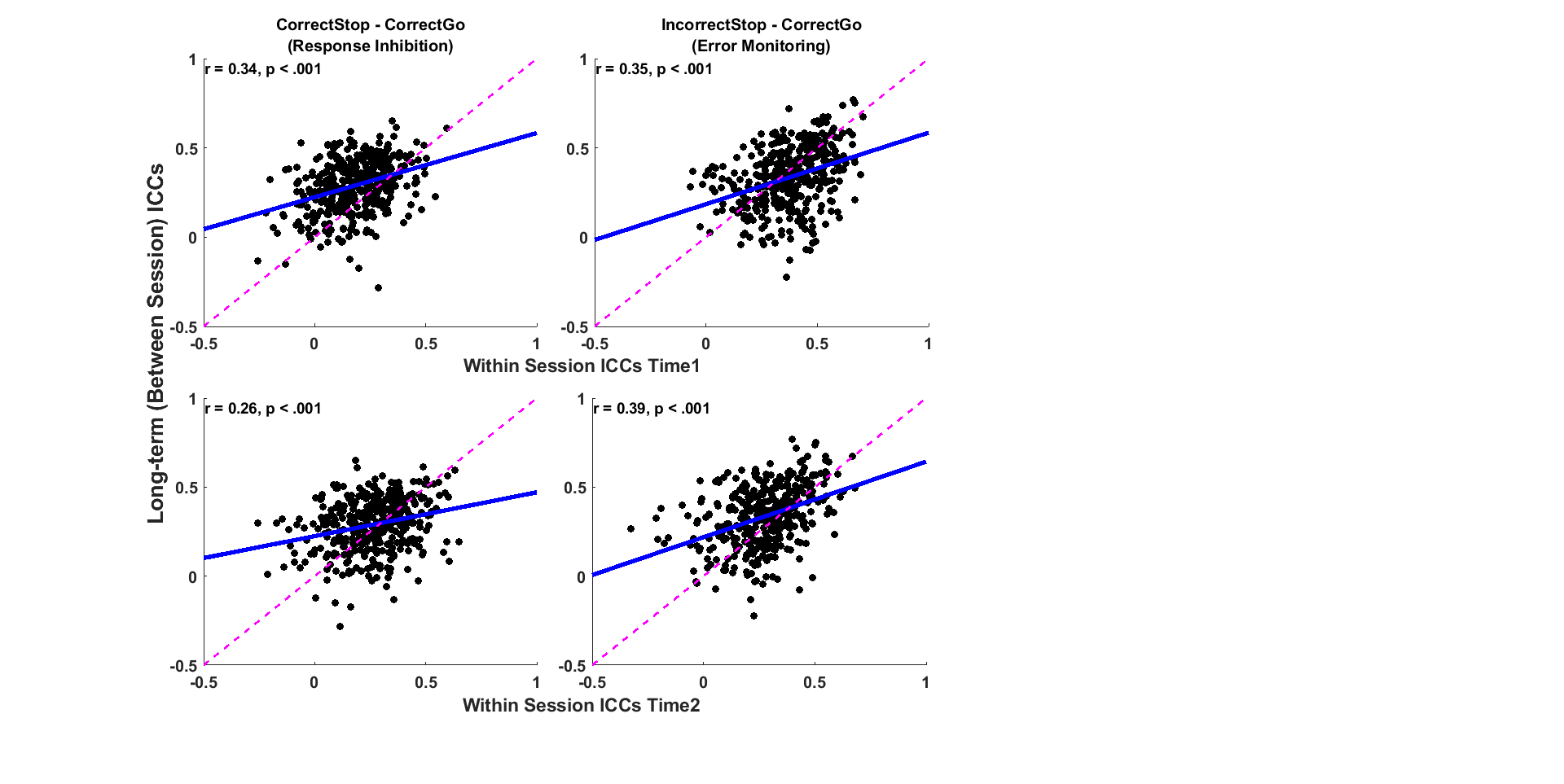
**

***Reliability across unrelated groups*.**

In order to investigate a potential bias in reliability estimates due to dependencies in the data (MZ twin correlations), reliabilities of the *unthresholded parcels* were re-estimated for two unrelated groups, by assigning Twin 1 and Twin 2 of our twin pairs to separate groups. This analysis revealed that reliabilities of the *unthresholded parcels* in the average of the two subsamples of unrelated individuals were highly correlated with those derived from the full sample (all *r*’s>.96, all *p’*s < .001).

**Figure S8.** Reliabilities of the *unthresholded parcels* in the average of two subsamples of unrelated individuals yielded values very similar to the full sample.

**
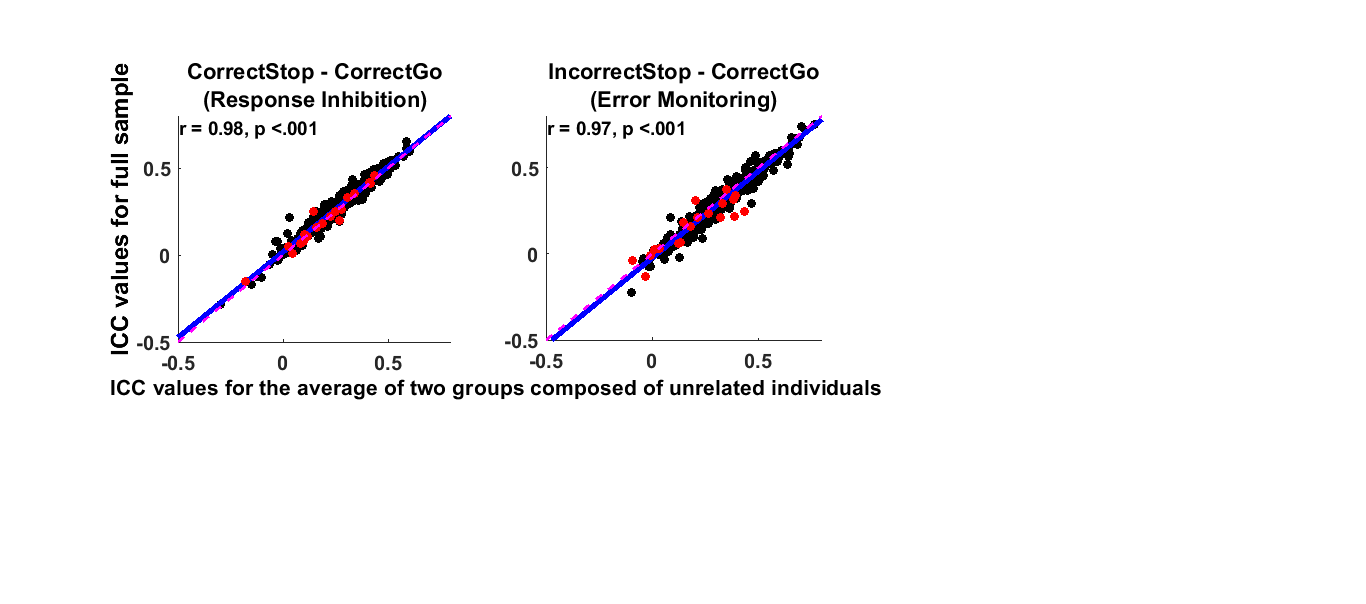
**

***Notes***. Each data-point represents a parcel/segment. **Black** markers represent cortical parcels and **red** markers represent subcortical segments. Magenta: line of identity, Blue: regression line.

**Figure S9.** After precleaning the BOLD data with multirun ICA-FIX, temporal Signal to Noise Ratio (tSNR) values increased for the *unthresholded* *parcels*.

***Unthresholded Parcels***


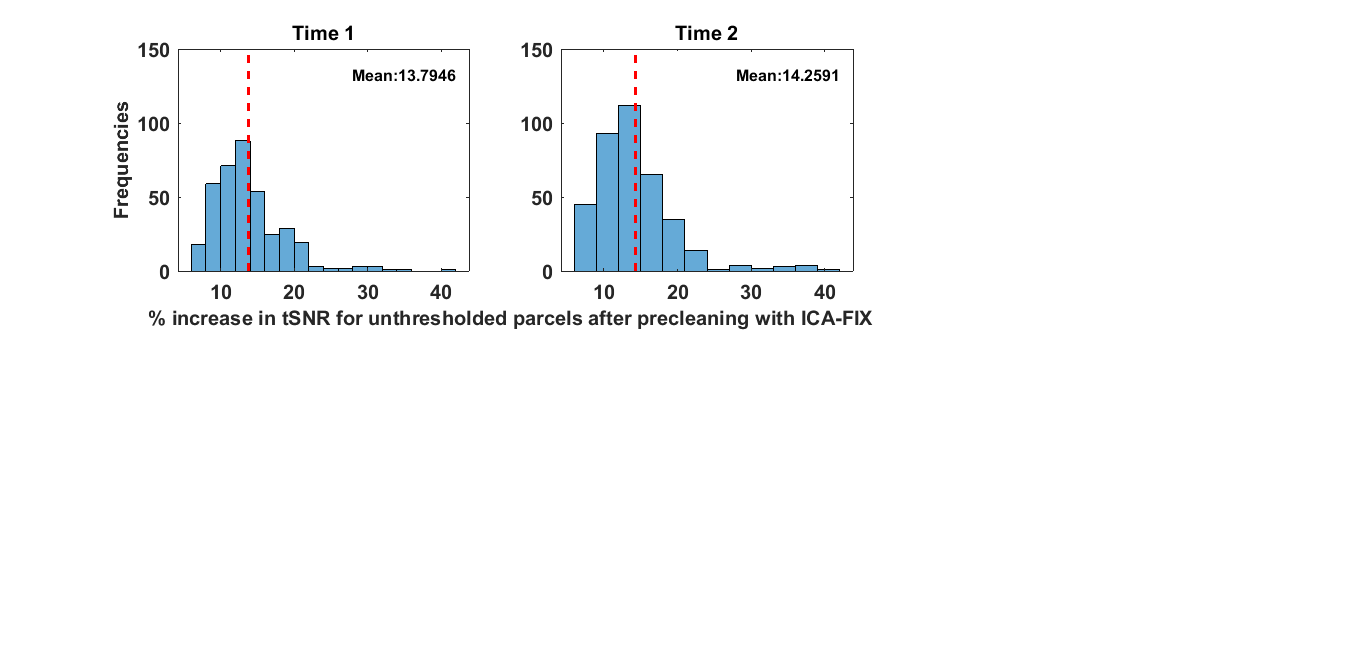


***Notes***. Temporal Signal to Noise Ratio (tSNR) for each grayordinate was calculated as the mean over time divided by the square root of the variance estimated from the residuals after model fitting (tfMRI *_Atlas.mean.dscalar.nii /√sigmasquareds.dtseries.nii) for each run separately, followed by taking the average across two runs per grayordinate. The averaged grayordinate-wise tSNR values were first parcellated (values across grayordinates within each parcel/segment were averaged) and then averaged across subjects (full sample of 56 subjects for Time1 and 44 subjects for Time1). For the *unthresholded parcels*, % increase in tSNR was calculated for the 360 cortical parcels and 19 subcortical segments. **Red** dashed line represents mean % increase in tSNRs across all 379 *unthresholded parcels/segments*.

**Table S1.** Test-retest reliabilities and twin correlations for the *unthresholded parcels* analysis. Only parcels that passed permutation-based significance testing for the test-retest ICCs are listed.

| **HCP-MP1.0 parcel name** | **Corresponding Desikan-Killiany Atlas** | **TRR ICC** | **Familiality** |
| --- | --- | --- | --- |
| **Correct Stop vs. Correct Go Contrast** **(Response Inhibition)**  Cutoff TRR ICC = .52; Cutoff familiality = .64 | | | |
| L_47s§ | Lateral orbitofrontal | 0.65 | 0.36 |
| R_47s§ | Lateral orbitofrontal | 0.61 | 0.23 |
| L_LO2§ | Lateral occipital | 0.61 | 0.15 |
| L_LO3§ | Lateral occipital | 0.59 | -0.02 |
| R_31a | Posterior cingulate | 0.57 | 0.40 |
| R_LO1§ | Lateral occipital | 0.56 | 0.17 |
| R_PH§ | Lateral occipital, fusiform, inferior temporal | 0.55 | 0.31 |
| R_V4§ | Lateral occipital, fusiform, lingual | 0.54 | 0.02 |
| R_IFJp§ | Caudal middle frontal, precentral | 0.53 | 0.41 |
| R_9p§ | Superior frontal, rostral middle frontal | 0.53 | 0.16 |
| R_IFSp§ | Pars opercularis, rostral middle frontal | 0.53 | 0.34 |
| L_43§ | Precentral | 0.53 | 0.09 |
| L_LO1§ | Lateral occipital | 0.53 | 0.07 |
| L_PoI1 | Insula | 0.52 | 0.07 |
| L_STV§ | Superior temporal, bankssts | 0.52 | 0.44 |
| R_LO2§ | Lateral occipital | 0.52 | 0.14 |
| R_DVT§ | Superior parietal, precuneus | 0.52 | 0.33 |
| **Incorrect Stop vs. Correct Go Contrast (Error Monitoring)**  Cutoff TRR ICC = .52; Cutoff familiality = .64 | | | |
| R_47s§ | Lateral orbitofrontal | 0.77 | 0.30 |
| R_9p§ | Superior frontal, rostral middle frontal | 0.75 | 0.47 |
| R_PFm§ | Inferior parietal, supramarginal | 0.74 | 0.54 |
| R_45§ | Pars opercularis, pars triangularis | 0.72 | -0.20 |
| R_PSL§ | Supramarginal, superior temporal | 0.67 | 0.19 |
| L_47s§ | Lateral orbitofrontal | 0.67 | 0.28 |
| R_a9_46v§ | Rostral middle frontal | 0.67 | 0.28 |
| R_LO1§ | Lateral occipital | 0.65 | 0.35 |
| R_STV§ | Superior temporal, bankssts | 0.65 | 0.47 |
| R_p47r§ | Rostral middle frontal, pars triangularis | 0.64 | 0.54 |
| L_9p§ | Superior frontal, rostral middle frontal | 0.64 | 0.40 |
| R_TPOJ1§ | Bankssts | 0.63 | 0.60 |
| R_9a§ | Superior frontal, rostral middle frontal | 0.61 | 0.34 |
| R_7PL§ | Superior parietal | 0.60 | 0.37 |
| L_p32 | Medial orbitofrontal, rostral anterior cingulate, superior frontal | 0.59 | 0.42 |
| L_8BL§ | Superior frontal | 0.59 | 0.69^a^ |
| R_IPS1§ | Superior parietal | 0.59 | 0.26 |
| R_FST§ | Middle temporal, lateral occipital, inferior temporal | 0.58 | 0.20 |
| R_8BL§ | Superior frontal | 0.58 | 0.47 |
| L_10r | Medial orbitofrontal | 0.58 | 0.38 |
| L_d32§ | Superior frontal, rostral anterior cingulate | 0.58 | 0.40 |
| R_V4t§ | Lateral occipital | 0.58 | 0.20 |
| R_PH§ | Lateral occipital, fusiform, inferior temporal | 0.58 | 0.35 |
| R_POS2§ | Precuneus, superior parietal | 0.58 | 0.57 |
| R_9_46d§ | Rostral middle frontal | 0.58 | 0.52 |
| R_LO2§ | Lateral occipital | 0.57 | 0.32 |
| R_MST§ | Inferior parietal, lateral occipital | 0.57 | 0.18 |
| R_8BM§ | Superior frontal | 0.57 | 0.32 |
| R_PF§ | Supramarginal | 0.57 | 0.14 |
| R_31pv | Istmuscingulate, precuneus | 0.57 | 0.37 |
| L_TPOJ1§ | Bankssts | 0.57 | 0.10 |
| L_V3B§ | Inferior parietal, superior parietal, lateral occipital | 0.57 | -0.07 |
|  |  |  |  |
| L_47l§ | Pars triangularis, pars orbitalis, lateral orbitofrontal | 0.57 | 0.18 |
| R_47l§ | Pars triangularis, pars orbitalis, lateral orbitofrontal | 0.56 | 0.40 |
| L_9m | Rostral anterior cingulate, superior frontal | 0.56 | 0.47 |
| L_PEF§ | Precentral | 0.56 | 0.13 |
| L_FST§ | Middle temporal, lateral occipital, inferior temporal | 0.56 | 0.32 |
| L_STV§ | Superior temporal, bankssts | 0.56 | 0.47 |
| R_p24§ | Rostral anterior cingulate | 0.55 | 0.56 |
| L_d23ab | Posterior cingulate, istmuscingulate | 0.55 | 0.26 |
| R_31pd§ | Precuneus | 0.55 | 0.12 |
| R_SFL§ | Superior frontal | 0.55 | 0.14 |
| R_8Av§ | Caudal middle frontal | 0.54 | 0.30 |
| R_IP0§ | Inferior parietal | 0.54 | 0.35 |
| R_TE1a§ | Middle temporal | 0.54 | 0.30 |
| R_MIP§ | Superior parietal | 0.54 | 0.35 |
| R_V7§ | Superior parietal | 0.54 | 0.25 |
| R_AAIC§ | Insula | 0.54 | 0.09 |
| R_PGi§ | Supramarginal, inferior parietal | 0.54 | 0.34 |
| L_LO1§ | Lateral occipital | 0.54 | 0.31 |
| R_d23ab | Posterior cingulate, istmus cingulate | 0.53 | 0.25 |
| L_IP0§ | Inferior parietal | 0.53 | -0.01 |
| L_a24 | Rostral anterior cingulate | 0.53 | 0.24 |
| L_p24§ | Rostral anterior cingulate | 0.53 | 0.27 |
| R_55b§ | Caudal middle frontal, precentral | 0.53 | 0.49 |
| L_11l§ | Rostral middle frontal, lateral orbitofrontal | 0.53 | 0.33 |
| R_d32§ | Superior frontal, rostral anterior cingulate | 0.52 | 0.14 |
| R_s6_8§ | Superior frontal | 0.52 | 0.19 |
| R_p32 | Medial orbitofrontal, rostral anterior cingulate, superior frontal | 0.52 | 0.22 |
| L_STSdp§ | Bankssts, superior temporal | 0.52 | 0.15 |
| R_VIP§ | Superior parietal | 0.52 | 0.02 |
| R_AIP§ | Superior parietal, supramarginal | 0.52 | 0.24 |
| R_TE2a§ | Inferior temporal, middle temporal | 0.52 | 0.50 |
| R_FEF§ | Caudal middle frontal, precentral | 0.52 | 0.60 |
|  |  |  |  |

***Notes****.* ^a^Regions with high test-retest reliability and MZ twin correlations, which are good candidate regions for phenotypes for future genetic and clinical studies (ICCs and rMZs are greater than or equal to the 95th quartile of their respective permutation distributions); Cutoff TRR ICC**:** the 95th quartile of the permuted ICC distribution. Cutoff familiality: the 95th quartile of the permuted MZ correlations. § parcels/segments that also contain significantly active grayordinates (grayordinate-wise FDR p< .05) during task performance (either entirely or partially within the parcel). Aside from the parcels reported in this table, parcel R_p47r (TRR ICC = .51; familiality = .66) and L_a9_46v (TRR ICC = .33; familiality = .66) in the*Correct Stop vs. Correct Go* contrast (response inhibition) had non-significant ICC values but significant familial effects (MZ twin correlations). R: right, L: left.

**Table S2.** Test-retest reliabilities and twin correlations for ROIs reported in the meta-analysis of Swick and colleagues (2011) for the successful inhibition events and Neta and colleagues (2015) for the unsuccessful inhibition events.

| **Correct Stop vs Correct Go Contrast (Response Inhibition)** | | | | | | |
| --- | --- | --- | --- | --- | --- | --- |
| **Region (Swick et al., 2011)** | **Coordinates** | **MMP1.0 Parcel/ FS segment** | Cutoff TRR ICC = .42; Cutoff familiality = .52 | **TRR ICC** | **TRR ICC**  **ICAFIX** | **Familiality** |
| L insula | -40,14,0 | L_PGP§ |  | .43* | .46* | .46 |
| R thalamus | 6,-20,0 | R thalamus§ |  | .12 | .30 | .005 |
| L putamen | -16,10,4 | L putamen |  | .33 | .27^a^ | .27 |
| R cingulate gyrus | 2,-24,28 | R_23d§ |  | .26 | .34 | .11 |
| R insula | 38,16,2 | R_MI§ |  | .19 | .28 | .03 |
| R inferior frontal gyrus | 44,12,22 | R_6r§ |  | .31 | .48* | .12 |
| R precentral gyrus | 42,4,34 | R_PEF |  | .32 | .44* | .32 |
| R medial frontal gyrus | 4,14,44 | R_p24pr§ |  | .23 | .34 | .12 |
| R middle frontal gyrus | 26,40,34 | R_9-46d¥§ |  | .38 | .42* | .40 |
| R inferior parietal lobule | 58,-40,26 | R_PSL§ |  | .38 | .48* | -.02 |
| R inferior parietal lobule | 48,-40,40 | R_IP2§ |  | .39 | .49* | .16 |
| R inferior parietal lobule | 34,-48,42 | R_AIP§ |  | .18 | .48* | .25 |
| R lentiform, Lateral GP | 14,6,0 | none |  | - | - | - |
| L superior temporal gyrus | -50,-40,16 | L_Pbelt§ |  | .20 | .38 | .04 |
| L middle temporal gyrus | -56,-50,6 | L_PHT§ |  | .44* | .40 ^a^ | .41 |
| R inferior occipital gyrus | 44,-70,-8 | R_FST§ |  | .45* | .49* | .09 |
| L superior frontal gyrus | -34,36,28 | L_46§ |  | .31 | .35 | .52* |
| R middle frontal gyrus | 28,-4,46 | R_8ad |  | .46* | .54* | .07 |
| R middle frontal | 30,0,54 | R_6a§ |  | .22 | .31 | .24 |
| L superior parietal | -24,-62,42 | L_MIP§ |  | .25 | .38 | .25 |
| L precentral gyrus | -40,4,32 | L_IFJp§ |  | .44* | .50* | .36 |
| L middle occipital | -36,-84,0 | L_LO2§ |  | .61* | .53* ^a^ | .15 |
| R superior temporal | 46,-26,0 | R_STSdp§ |  | **.39** | **.42*** | **.59*** |
| R superior parietal | 26,-56,46 | R_LIPv§ |  | .43* | .47* | .18 |
| **MEAN** |  |  |  | **.34** | **.42** |  |
| **Incorrect Stop vs Correct Go Contrast (Error Monitoring)** | | | | | | |
| **Region (Neta et al., 2015)** | **Coordinates** | **MMP1.0 Parcel/ FS segment** | Cutoff TRR ICC = .38 familiality =.48 | **TRR ICC** | **TRR ICC**  **ICAFIX** | **Familiality** |
| L anterior insula/frontal operculum | -35,18,8 | L_FOP4§ |  | .47* | .40 ^a^ | .40 |
| R anterior insula/frontal operculum | 35,23,4 | R_FOP4§ |  | .49* | .38 ^a^ | .20 |
| L medial superior frontal (dorsal) | -6,11,51 | L_SCEF§ |  | .26 | .44* | .31 |
| R anterior insula/frontal operculum (lateral) | 46,22,3 | R_44§ |  | .42* | .38 ^a^ | -.01 |
| R medial superior frontal (middle) | 6,25,36 | R_a32pr§ |  | .50* | .23 ^a^ | .22 |
| R medial superior frontal (dorsal) | 6,18,44 | R_8BM§ |  | .57* | .52* ^a^ | .32 |
| L medial superior frontal (middle) | -6,21,42 | L_8BM§ |  | .14 | .32 | .35 |
| R medial superior frontal (ventral) | -5,30,32 | L_d32§ |  | .58* | .48* ^a^ | .40 |
| R dorsolateral prefrontal | 42,7,40 | R_55b§ |  | **.53*** | **.55*** | **.49*** |
| L dorsolateral prefrontal | -45,14,29 | L_IFJa |  | .32 | .24 ^a^ | .05 |
| Caudate L | -12,7,8 | caudate |  | .37 | .24 ^a^ | .31 |
| **MEAN** |  |  |  | **.42** | **.38** |  |

***Notes****.* ^*^Regions with significant test-retest reliability and MZ twin correlations (ICCs and rMZs are greater than or equal to the 95th quartile of their respective permutation distributions). Cutoff TRR ICC**:** the 95th quartile of the permuted ICC distribution. Cutoff familiality: the 95th quartile of the permuted MZ correlations. **Bold**: Regions with significant test-retest reliability and MZ twin correlations, which are good candidate regions for phenotypes for future genetic and clinical studies. TRR ICC ICAFIX Column: ICC estimates after cleaning the fMRI data with the multirun ICA-FIX method.  ^a^ Regions that showed similar or lower ICCs after multirun ICA–FIX cleaning. § parcels/segments that also contain significantly active grayordinates (grayordinate-wise FDR p< .05) during task performance (either entirely or partially within the parcel). ¥: parcels that overlap with DLPFC.

**RESULTS FOR TASK-RELATED GROUP LEVEL ACTIVATIONS (*THRESHOLDED PARCELS ANALYSIS*)**

**Data Analysis and Definition of Brain Regions**

We identified a set of regions showing the strongest task-related group level activations in our dataset (referred to as *thresholded parcels* analysis) because activation data from previous studies may not fully generalize to the present data due to differences in task design and analytical pipelines.

For the *thresholded parcel* analysis, group level grayordinate-wise statistical maps were created separately for Time1 and Time2 data by using permutation statistics as implemented in the PALM toolbox, version alpha101 (Permutation Analysis of Linear Models, <http://fsl.fmrib.ox.ac.uk/fsl/fslwiki/PALM>, (Winkler et al., 2014)), and using all available participants (n=56 at Time1 and n=44 at Time2). Multi-level exchangeability blocks (Winkler et al., 2015), which limit the permutations within block level (i.e., between two MZ siblings), were used to account for the shared variance between twins. We did not correct for the multiplicity of contrasts.

For the *thresholded parcels* analysis, the whole-brain grayordinate-wise FDR corrected maps were used to define significant clusters for each contrast as groups of spatially contiguous grayordinates exceeding 80mm^2^/120 mm^3^ (surface/volume for cortical and subcortical regions, respectively). 80mm^2^ and 120 mm^3^ correspond to projection of 20 voxels (2mm x 2mm x 20voxels) on the surface for cortical regions and 15 voxels of volume (2mm x 2mm x 2mm x 15voxels) for subcortical structures, respectively. These values were selected upon visual inspection of cluster extent in activation maps and also taking into account that the cortical areas by definition are larger as compared to subcortical structures. The FDR corrected significant clusters were further divided into anatomical parcels (286 and 257 parcels/segments in the *Correct Stop vs Correct Go* and *Incorrect Stop vs Correct Go* contrasts, respectively). Throughout the text, the term “*thresholded parcels*” is used to refer to the conjunction between a parcel/segment and the FDR corrected significant clusters. Thus, all thresholded activations respected the parcellation boundaries (and were entirely inside one of the parcels), but only contained above threshold vertices/voxels.

Outlier exclusion criteria described in the main text was also applied to the thresholded parcels. With this exclusion procedure, from the Time1 and Time2 data together, 0.81% and 0.51% of data-points from the *thresholded parcels* mean BOLD data in the *Correct Stop vs Correct Go* (Response Inhibition) and *Incorrect Stop vs Correct Go* (Error Monitoring) contrasts, respectively; were replaced with missing values. From the fMRI data (beta weights) cleaned by MR-ICA-FIX, 0.46% and 0.62% of data-points from the *thresholded parcels* mean BOLD data were excluded, respectively, for Time1 and Time2.

Note that the group-level activation maps were based on the data without MR-ICA-FIX cleaning.

**Figure S10.** Regions with significant brain activations in the *Correct Stop vs Correct Go* (Response Inhibition) and *Incorrect Stop vs Correct Go* (Error Monitoring) contrasts at Time1 and Time2 (masked by grayordinate-wise FDR p< .05 maps, computed via permutation testing using the PALM toolbox). [Data, including the specific regions in the *thresholded parcels,* can be found in the BALSA repository for neuroimaging data: <https://balsa.wustl.edu/study/show/wN9n4>].


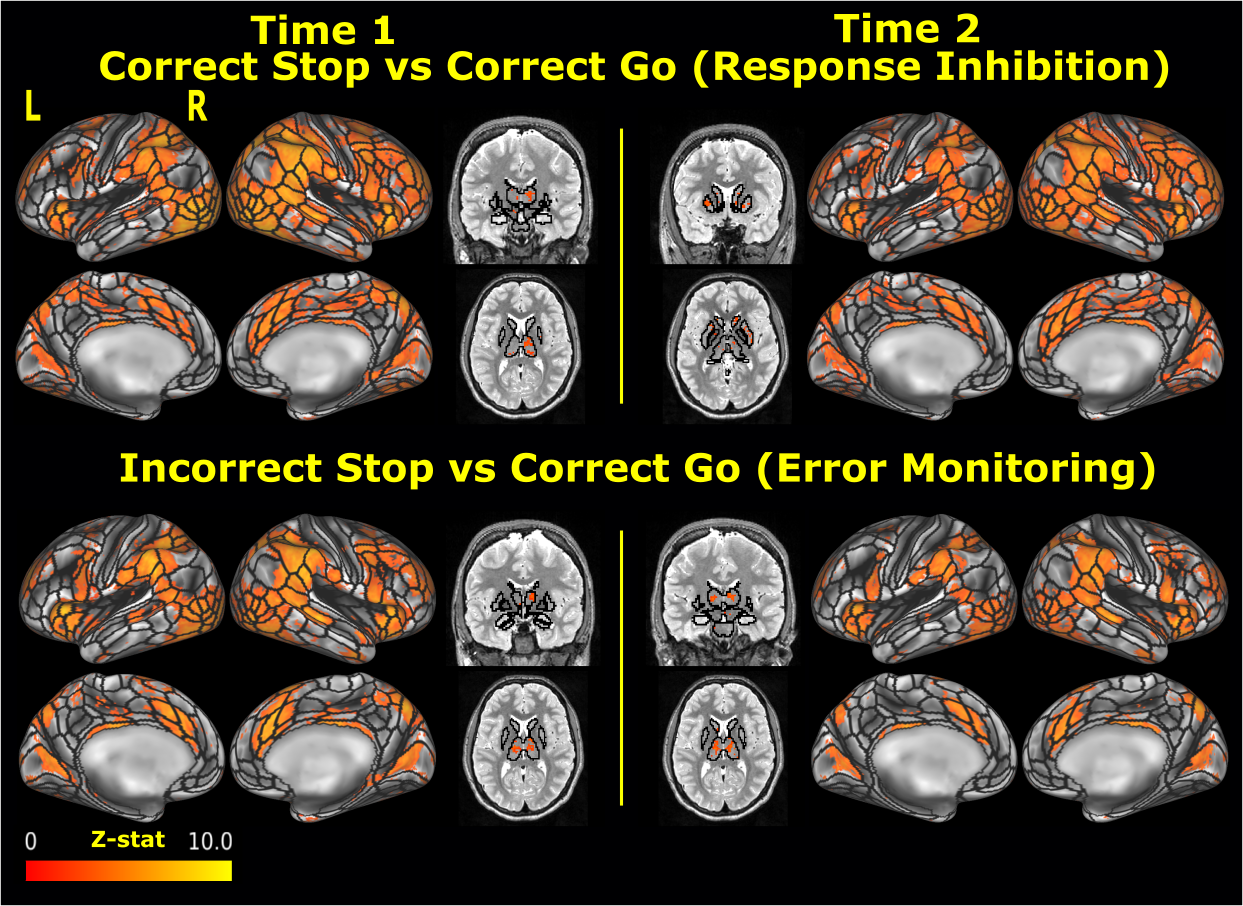


***Notes***. L: left, R: right.

**Figure S11.** Distribution of maximum ICC across permutations from the *thresholded parcels* analysis.

**
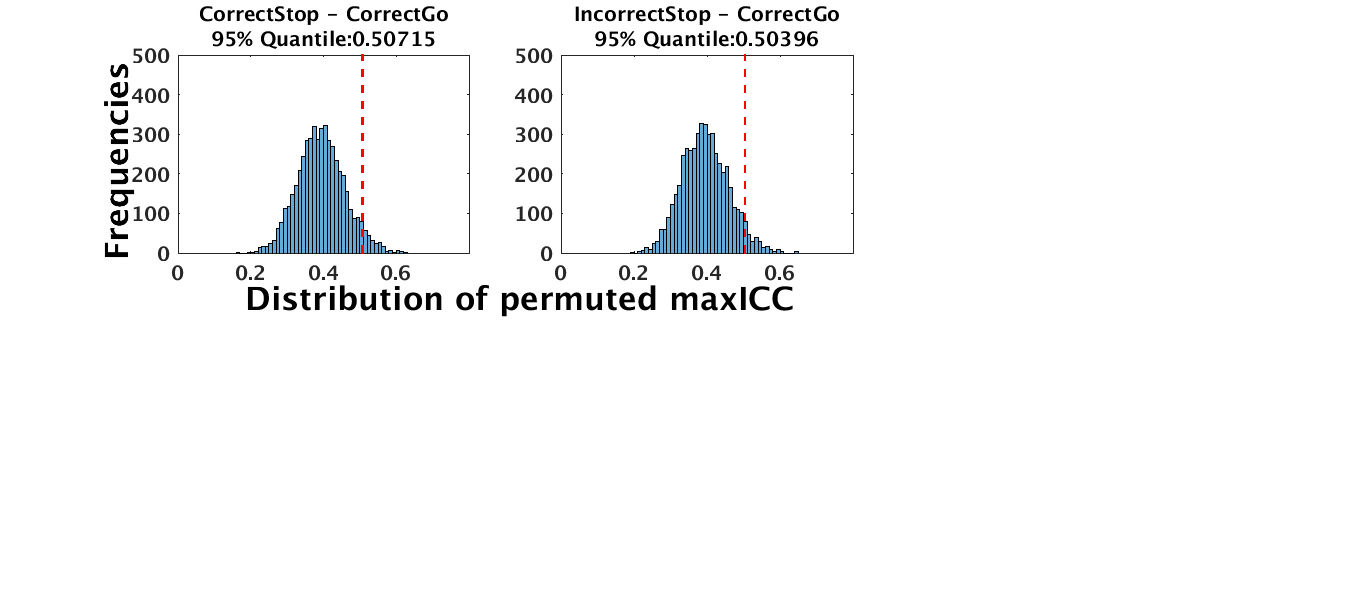
**

***Notes***. For details, see Figure S2 notes.

**Figure S12.** Distribution of permuted twin correlations from the *thresholded parcels* analysis.

**
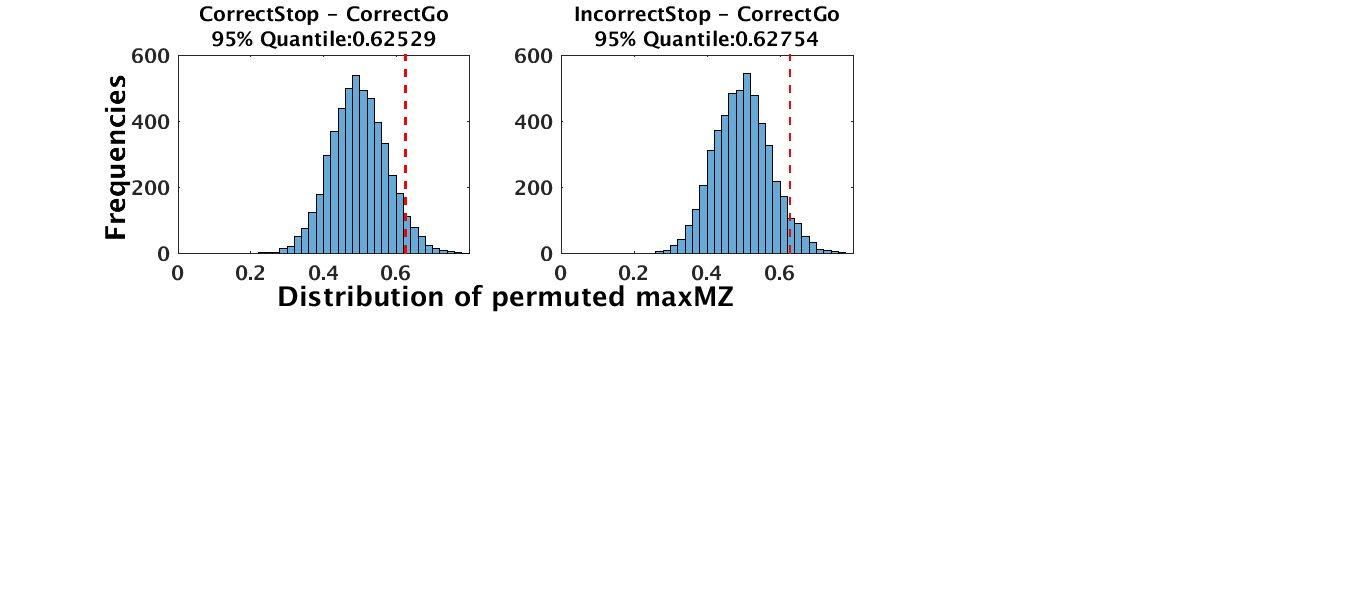
**

***Notes***. For details, see figure S5 notes.

**Test-retest reliability**

*Reliability of inhibition-related activity*.

Within the broad task related active regions (*thresholded parcels)* (see Table S3 and Figure S13)*,* significant ICC values ranged from .51 to .64 for the *Correct Stop vs Correct Go* contrast. The left pars triangularis/orbitalis, right lateral orbitofrontal, right rostral and caudal middle frontal regions showed good reliabilities in the successful inhibition versus successful activation trials.

*Reliability of error-related activity*.

Within the broad task related active regions (*thresholded parcels)* (see Table S3 and Figure S13)*,* significant ICC values ranged from .50 to .75 for the *Incorrect Stop versus Correct Go* contrast. The superior frontal, pars triangularis/opercularis, lateral orbitofrontal, rostral anterior cingulate, caudal middle frontal, and rostral middle frontal showed good reliabilities.

**Figure S13.** **Test-retest reliabilities (ICCs, left) and Familiality (right) of significant task-related activations varied from none-to-high across different parcels (thresholded parcels analysis)**. The FDR corrected significant clusters were further divided into anatomical segments using the Human Connectome Project Multi-Modal Parcellation (MMP1.0) and the Freesurfer subcortical segmentation. ICCs were mapped separately for the *Correct Stop vs Correct Go* and *Incorrect Stop vs Correct Go* contrasts. On the cortical surface view, *Black* outlines depict the boundaries of the MMP1.0 cortical parcellation. The bilateral caudate, cerebellum, left accumbens, right thalamus and right hippocampus in the *Correct Stop vs Correct Go* contrast and the bilateral thalamus, hippocampus and right cerebellum in the *Incorrect Stop vs Correct Go* contrast had significant activations, however none of the subcortical regions has significant ICCs and are not depicted in this figure. Cortical parcels with negative ICC values are not plotted.

**
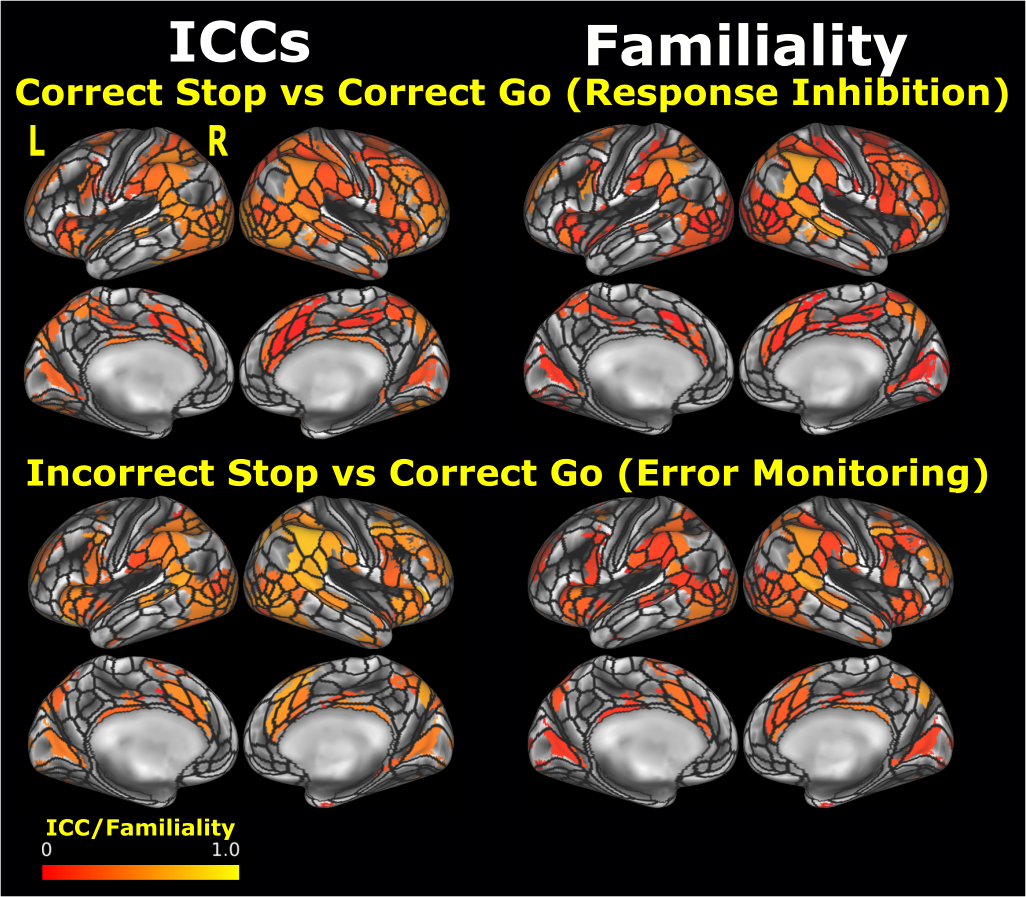
**

***Notes***. L: left, R: right.

**Table S3.** Test-retest reliabilities and twin correlations for task related active regions (*thresholded parcels)*. Only parcels that passed permutation-based significance testing (p<0.05) for the test-retest ICCs are listed.

| **HCP-MP1.0 parcel name** | **Corresponding Desikan-Killiany Atlas** | **TRR ICC** | **Familiality** |
| --- | --- | --- | --- |
| **Correct Stop vs Correct Go Contrast (Response Inhibition)**  Cutoff TRR ICC = .51; Cutoff familiality = .63 | | | |
| L_PGi | Supramarginal, inferior parietal | 0.64 | 0.22 |
| L_LO2 | Lateral occipital | 0.61 | 0.15 |
| L_47l | Pars triangularis, pars orbitalis, lateral orbitofrontal | 0.61 | 0.04 |
| L_LO3 | Lateral occipital | 0.59 | -0.02 |
| R_47s | Lateral orbitofrontal | 0.58 | 0.36 |
| L_STSdp | Bankssts, superior temporal | 0.56 | 0.30 |
| R_LO1 | Lateral occipital | 0.56 | 0.17 |
| R_a946v¥ | Rostral middle frontal | 0.56 | 0.42 |
| R_p47r | Rostral middle frontal, pars triangularis | 0.55 | 0.65* |
| L_TF | Fusiform, inferior temporal | 0.55 | 0.19 |
| R_V4 | Lateral occipital, fusiform, lingual | 0.55 | 0.02 |
| R_PH | Lateral occipital, fusiform, inferior temporal | 0.55 | 0.31 |
| R_IFJp | Caudal middle frontal, precentral | 0.54 | 0.40 |
| R_PI | Superior temporal | 0.54 | -0.09 |
| L_LO1 | Lateral occipital | 0.53 | 0.07 |
| R_LO2 | Lateral occipital | 0.52 | 0.15 |
|  |  |  |  |
| L_FST | Middle temporal, lateral occipital, inferior temporal | 0.51 | 0.04 |
| R_V3 | Superior parietal, lateral occipital, lingual | 0.51 | -0.12 |
| L_TPOJ1 | Bankssts | 0.51 | -0.10 |
| **Incorrect Stop vs Correct Go Contrast (Error Monitoring)**  Cutoff TRR ICC = .50; Cutoff familiality = .63 | | | |
| R_9a | Superior frontal, rostral middle frontal | 0.75 | 0.46 |
| R_PFm | Inferior parietal, supramarginal | 0.74 | 0.54 |
| R_45 | Pars opercularis, pars triangularis | 0.72 | -0.28 |
| R_47s | Lateral orbitofrontal | 0.71 | 0.39 |
| R_9p | Superior frontal, rostral middle frontal | 0.70 | 0.45 |
| L_47s | Lateral orbitofrontal | 0.70 | 0.39 |
| R_PGi | Supramarginal, inferior parietal | 0.68 | 0.50 |
| L_d32 | Superior frontal, rostral anterior cingulate | 0.67 | 0.19 |
| R_PSL | Supramarginal, superior temporal | 0.67 | 0.19 |
| R_STV | Superior temporal, bankssts | 0.65 | 0.46 |
| R_TPOJ1 | Bankssts | 0.64 | 0.59 |
| L_47l | Pars triangularis, pars orbitalis, lateral orbitofrontal | 0.64 | -0.02 |
| R_FEF | Caudal middle frontal, precentral | 0.63 | 0.45 |
| L_V3B | Inferior parietal, superior parietal, lateral occipital | 0.63 | -0.02 |
| L_PGi | Supramarginal, inferior parietal | 0.62 | 0.02 |
| R_IPS1 | Superior parietal | 0.62 | 0.30 |
| R_POS2 | Precuneus, superior parietal | 0.62 | 0.59 |
| R_946d¥ | Rostral middle frontal | 0.61 | 0.52 |
| R_55b | Caudal middle frontal, precentral | 0.61 | 0.59 |
| R_SCEF | Superior frontal | 0.61 | 0.45 |
| L_TPOJ1 | Bankssts | 0.61 | 0.11 |
| R_p47r | Rostral middle frontal, pars triangularis | 0.60 | 0.09 |
| R_7PL | Superior parietal | 0.60 | 0.38 |
| R_a946v¥ | Rostral middle frontal | 0.59 | 0.25 |
| L_11l | Rostral middle frontal, lateral orbitofrontal | 0.59 | 0.42 |
| R_PF | Supramarginal | 0.59 | 0.15 |
| R_PGs | Inferior parietal | 0.58 | 0.38 |
| R_LO1 | Lateral occipital | 0.58 | 0.35 |
| R_PH | Lateral occipital, fusiform, inferior temporal | 0.58 | 0.32 |
| R_8BM | Superior frontal | 0.58 | 0.35 |
| R_V4t | Lateral occipital | 0.58 | 0.20 |
| R_LO2 | Lateral occipital | 0.57 | 0.32 |
| R_MST | Inferior parietal, lateral occipital | 0.57 | 0.18 |
| R_8Av | Caudal middle frontal | 0.56 | 0.18 |
| L_FST | Middle temporal, lateral occipital, inferior temporal | 0.56 | 0.32 |
| L_PEF | Precentral | 0.55 | 0.11 |
| R_V2 | Lingual, lateral occipital, cuneus | 0.55 | 0.08 |
| R_IP0 | Inferior parietal | 0.55 | 0.32 |
| L_a946v¥ | Rostralmiddlefrontal | 0.55 | 0.08 |
| R_V3B | Inferior parietal, superior parietal, lateral occipital | 0.55 | 0.25 |
| R_V7 | Superior parietal | 0.55 | 0.26 |
| R_SFL | Superior frontal | 0.55 | 0.14 |
| R_31pd | Precuneus | 0.55 | 0.10 |
| R_MIP | Superior parietal | 0.54 | 0.35 |
| R_d32 | Superior frontal, rostral anterior cingulate | 0.54 | 0.18 |
| R_MI | Insula | 0.54 | 0.27 |
| R_TE2a | Inferior temporal, middle temporal | 0.54 | 0.33 |
| R_AAIC | Insula | 0.54 | 0.10 |
| R_A5 | Bankssts | 0.54 | 0.25 |
| R_8BL | Superior frontal | 0.54 | 0.12 |
| R_FST | Middle temporal, lateral occipital, inferior temporal | 0.54 | 0.46 |
| L_LO1 | Lateral occipital | 0.54 | 0.31 |
| R_IFJa | Caudal middle frontal, pars opercularis | 0.54 | 0.33 |
| L_8C | rostral middle frontal, caudal middle frontal | 0.53 | 0.06 |
| R_i68 | Caudal middle frontal | 0.53 | 0.22 |
| R_AIP | Superior parietal, supramarginal | 0.53 | 0.16 |
| L_IP0 | Inferior parietal | 0.53 | -0.06 |
| L_STV | Superior temporal, bankssts | 0.52 | 0.45 |
| L_STSdp | Bankssts, superior temporal | 0.52 | 0.23 |
| R_TE2p | Inferior temporal | 0.52 | 0.32 |
| L_STSvp | Bankssts, middle temporal | 0.52 | 0.06 |
| R_PFop | Postcentral, supramarginal | 0.51 | 0.44 |
| L_IP1 | Inferior parietal | 0.51 | -0.05 |
| L_LO2 | Lateral occipital | 0.51 | 0.28 |
| R_8C | Rostral middle frontal, caudal middle frontal | 0.50 | 0.16 |
| R_a32pr | Superior frontal, caudal anterior cingulate, superior frontal | 0.50 | 0.22 |
| R_TE1a | Middle temporal | 0.50 | 0.16 |

***Notes****.* ^*^Regions with significant MZ twin correlations (rMZs are greater than or equal to the 95th quartile of their respective permutation distributions, note that all regions reported here had significant ICC estimates), which are good candidate regions for phenotypes for future genetic and clinical studies. Cutoff TRR ICC**:** the 95th quartile of the permuted ICC distribution. Cutoff familiality: the 95th quartile of the permuted MZ correlations. ¥: parcels that overlap with the DLPFC. Although significant FDR corrected group level subcortical activations were observed in both conditions, none of these regions had significant ICC values. Aside from the parcels reported in this table, there were no other *thresholded parcels* that had non-significant ICC values but significant familial effects (MZ twin correlations). R: right, L: left.

**Figure S14. After precleaning the BOLD data with multirun ICA-FIX, temporal Signal to Noise Ratio (tSNR) values increased for the *thresholded* *parcels*.**

***Thresholded Parcels***

**
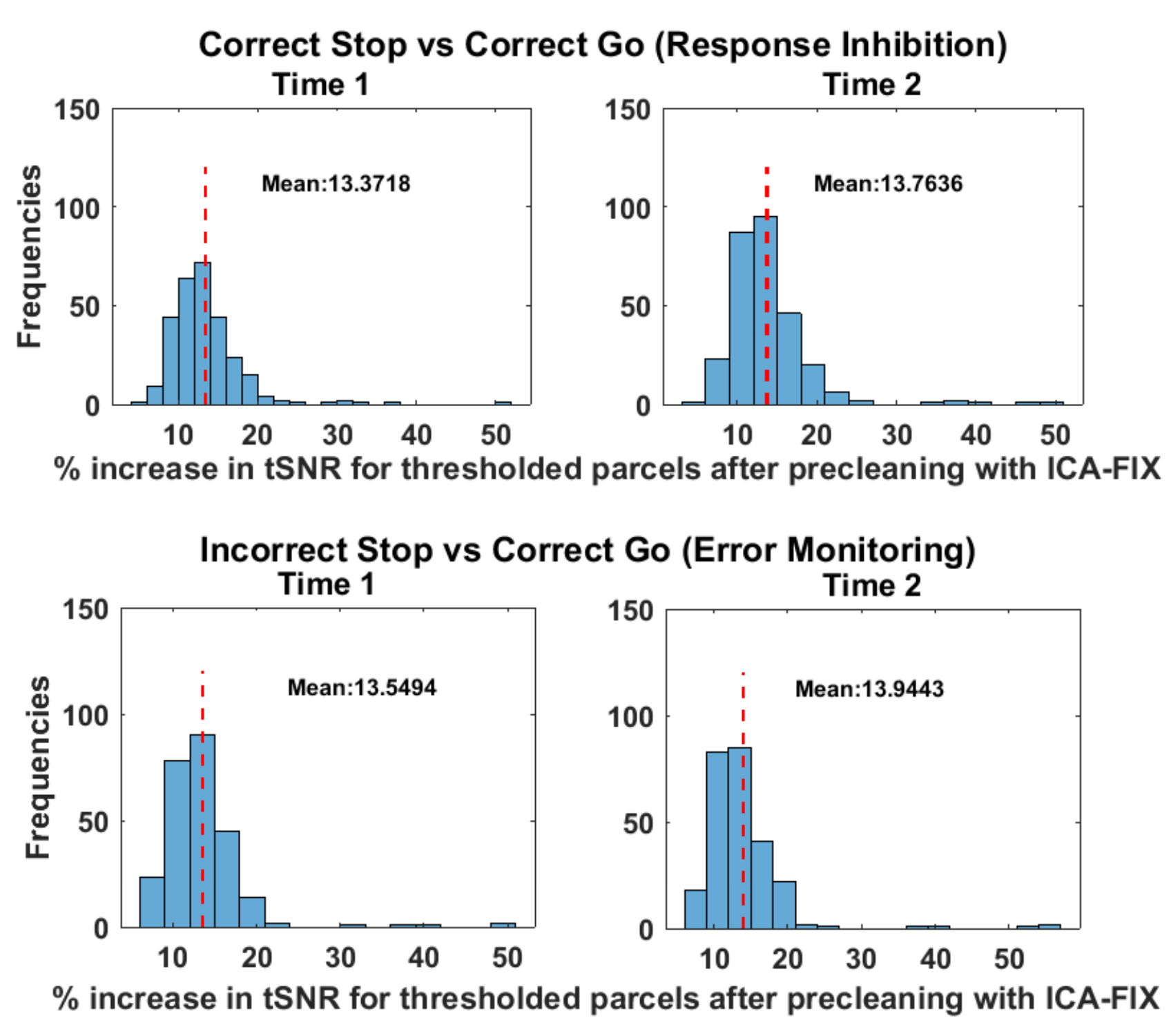
**

***Notes***. For details, see Figure S9 notes. For the *thresholded parcels*, % increases in tSNRs were plotted for the parcels/segments that showed significant FDR corrected activations and are displayed separately here per contrast. **Red** dashed line represents mean % increase in tSNRs across all *thresholded parcels* (286 parcels/segments in the *CorrectStop vs CorrectGo* contrast and 257 parcels/segments in the *IncorrectStop vs CorrectGo* contrast).

***Does ICA-based artifact removal improve the test-retest reliability of brain activations?***

Figure S15 depicts the distribution of ICC estimates across the activated regions (thresholded parcels). ICC estimates were slightly greater after cleaning the data with multirun ICA-FIX for the *Correct Stop vs Correct Go* contrast*,* but slightly lower for the *Incorrect Stop vs Correct Go* contrast (average difference, respectively are 0.07, and -0.02) across *thresholded parcels* (paired t-test *p*s < .001 and .03, respectively).

**Figure S15. ICC estimates slightly improved for activation data (thresholded parcels) after cleaning the data with multirun ICA-FIX for inhibition but not for error monitoring.** The distribution of ICCs before and after cleaning the data with multirun ICA-FIX, for the unthresholded parcels/segments (upper panel) and histogram of differences in ICCs before and after cleaning the data with multirun ICA-FIX (lower panel).

**
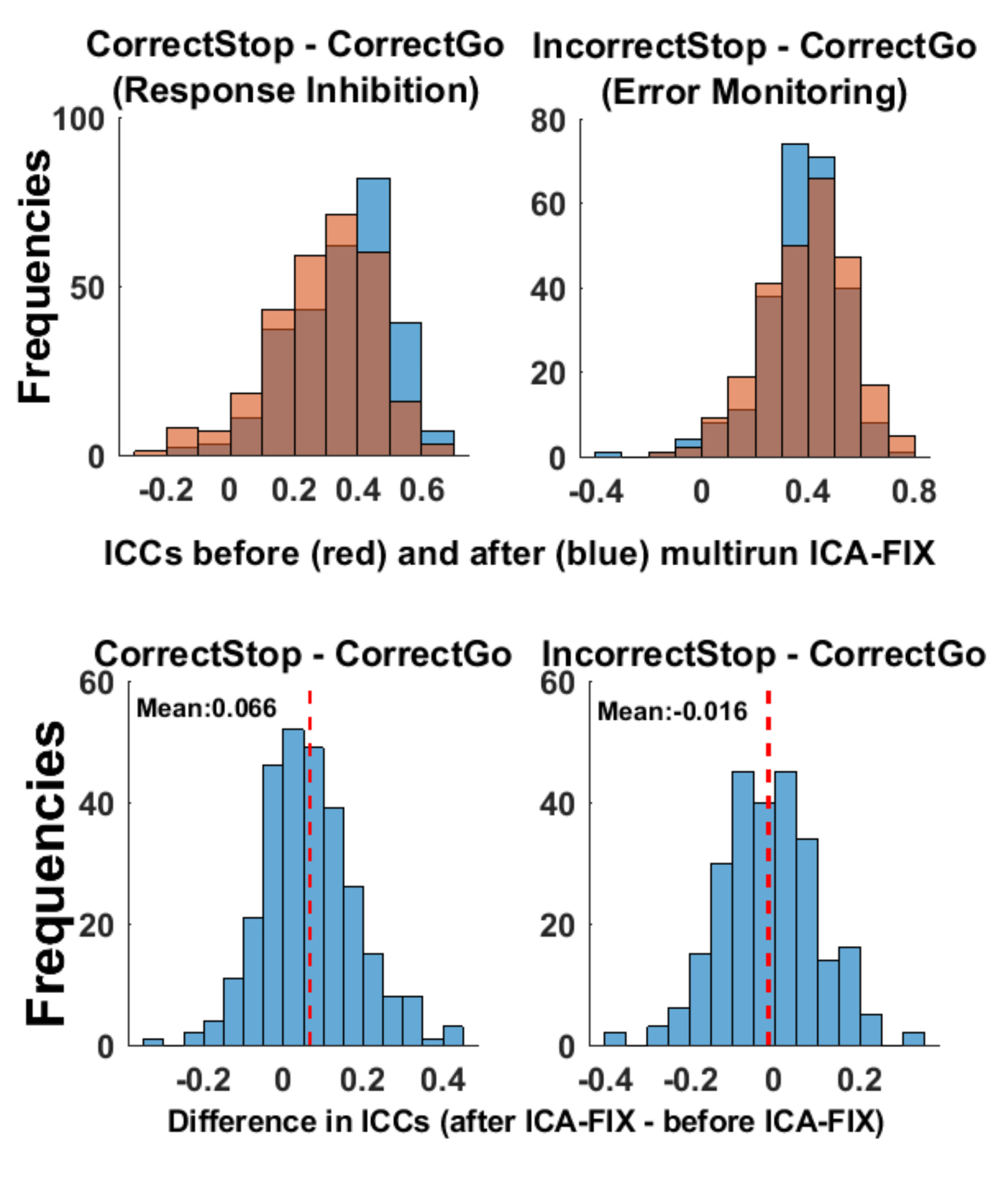
**

***Notes***. **Red** dashed line represents mean difference in ICCs across all *thresholded parcels* (286 parcels/segments in the *CorrectStop vs CorrectGo* contrast and 257 parcels/segments in the *IncorrectStop vs CorrectGo* contrast).

**REFERENCES**

Chapman, H.A., Bernier, D., Rusak, B. (2010). MRI-related anxiety levels change within and between repeated scanning sessions. Psychiatry Res. Neuroimaging 182, 160–164. [doi.org/10.1016/j.pscychresns.2010.01.005](https://doi.org/10.1016/j.pscychresns.2010.01.005)

Glasser, M.F., Coalson, T.S., Robinson, E.C., Hacker, C.D., Harwell, J., Yacoub, E., et al. (2016). A multi-modal parcellation of human cerebral cortex. Nature 536, 171–178. [doi.org/10.1038/nature18933](https://doi.org/10.1038/nature18933)

Marcus, D.S., Harwell, J., Olsen, T., Hodge, M., Glasser, M.F., Prior, F., et al. (2011). Informatics and data mining tools and strategies for the Human Connectome Project. Front. Neuroinform. 5, 1–12. [doi.org/10.3389/fninf.2011.00004](https://doi.org/10.3389/fninf.2011.00004)

Winkler, A.M., Ridgway, G.R., Webster, M.A., Smith, S.M., Nichols, T.E. (2014). Permutation inference for the general linear model. Neuroimage 92, 381–397.

Winkler, A.M., Webster, M.A., Vidaurre, D., Nichols, T.E., Smith, S.M. (2015). Multi-level block permutation. Neuroimage 123, 253–268.
